# Emerging Role of Nb_2_CT_x_ MXene in Sensors: The Roadmap from Synthesis to Health and Environmental Monitoring

**DOI:** 10.3390/s25123691

**Published:** 2025-06-12

**Authors:** Gyu Jin Choi, Jeong Won Ryu, Hwa Jun Jeon, Rajneesh Kumar Mishra, Yoonseuk Choi, Jin Seog Gwag

**Affiliations:** 1 Department of Physics, Yeungnam University, Gyeongsan 38541, Gyeongbuk, Republic of Korea; gyujin1125@gmail.com (G.J.C.); rjw7005@naver.com (J.W.R.); d_lab@ynu.ac.kr (H.J.J.); 2 Department of Electronics and Control Engineering, Hanbat National University, Yuseong-gu, Daejeon 34158, Republic of Korea; ychoi@hanabt.ac.kr

**Keywords:** Nb_2_CT_x_ MXene, functionalization, surface termination tuning, properties, gas sensors

## Abstract

The rise of two-dimensional (2D) materials has transformed gas sensing, with Nb_2_CT_x_ MXene drawing significant interest due to its distinct physicochemical behaviors. As part of the MXene family, Nb_2_CT_x_ MXene demonstrates a remarkable combination of high electrical conductivity, adjustable surface chemistry, and exceptional mechanical flexibility, positioning it as a promising candidate for next-generation gas sensors. This review explores the synthesis techniques for Nb_2_CT_x_ MXene, highlighting etching methods and post-synthesis adjustments to achieve the tailored surface terminations and structural qualities essential for gas detection. A comprehensive examination of the crystal structure, morphology, and electronic characteristics of Nb_2_CT_x_ MXene is presented to clarify its outstanding sensing capabilities. The application of Nb_2_CT_x_ MXene for detecting gases, including NH_3_, humidity, NO_2_, and volatile organic compounds (VOCs), is assessed, showcasing its sensitivity, selectivity, and low detection limits across various environmental settings. Furthermore, the integration of Nb_2_CT_x_ MXene with other nanostructures in sensor platforms is reviewed. Lastly, challenges related to scalability, stability, and long-term performance are addressed, along with future prospects for Nb_2_CT_x_ MXene-based gas sensors. This review offers significant insights into the potential of Nb_2_CT_x_ MXene as a pioneering material for enhancing gas sensing technologies.

## 1. Introduction

Detecting hazardous gases depends on sensors to monitor various environments, ensuring safety and environmental protection [1]. Toxic gas levels continue to rise due to industrial activities, urban development, and vehicle emissions, which release CO, NO_x_, SO_2_, NH_3_, and VOCs [2]. The emission of dangerous gases into the atmosphere contributes to several health issues affecting respiration, heart function, and the nervous system [3]. In industrial areas, explosive gases like hydrogen (H_2_) and methane (CH_4_) require monitoring to prevent serious industrial accidents [4]. Within healthcare institutions, gas sensors are diagnostic tools for detecting respiratory substances and identifying biomolecular indicators for diabetes and lung cancer [5]. Gas sensors enable the food packaging industry to maintain product quality by continuously measuring gas composition [6]. Various industries demand precise gas detection systems, which motivates researchers to advance the development of more selective and reliable gas sensing technologies [7]. Traditional gas sensors have achieved significant milestones but face several challenges that limit their widespread use [8]. Cross-sensitivity occurs because many gas sensors struggle to differentiate between similar chemical structures [9]. The operation of metal oxide semiconductor (MOS) sensors requires high temperatures ranging from 200 °C to 400 °C, resulting in significant power consumption issues [10].

Currently, sensors used for gas detection exhibit limited operational capacities due to slow adsorption and desorption processes [11]. Gas sensors experience diminished performance over time due to aging materials, exposure to humidity, and contamination effects [12]. Using specific gas sensors becomes economically unfeasible since their production requires intricate processing methods and expensive components [13]. Pursuing miniaturized portable devices presents challenges in developing compact, high-performance sensing elements within gas sensor technologies [14]. The inadequate capabilities of existing materials highlight the urgency for new sensing solutions utilizing two-dimensional (2D) materials, as these offer three key advantages: increased surface area, adjustable electronic properties, and exceptional chemical resistance [15]. Various materials have been explored for gas sensing applications, with each type presenting unique advantages and limitations [16]. Metal oxide semiconductors (MOSs) such as ZnO, SnO_2_, TiO_2_, and WO_3_ function through resistance variations during gas adsorption and desorption [17]. However, these materials are limited by their requirement for high operating temperatures and the inability to detect multiple gases selectively [18]. Conducting polymers, such as polyaniline (PANI), polypyrrole (PPy), and polythiophene (PTh), serve as effective gas sensing materials due to their tunable conductivity, straightforward fabrication methods, and low operating temperatures [19]. Nevertheless, their long-term stability remains a significant issue [20]. Carbon-derived nanomaterials, including graphene, carbon nanotubes, and graphene oxide, display an exceptional surface area, excellent electrical properties, and high gas sensitivity [21]. However, these materials struggle to detect specific targets and require complex functionalization processes to be selectively applied [22].

The emerging two-dimensional materials known as MXenes, comprised of transition metal carbides and nitrides, have attracted considerable research interest due to their metallic conductivity, hydrophilic nature, and diverse surface functionalities [23]. MXenes form a rapidly expanding family of 2D materials derived from the selective extraction of “A” elements in MAX phase layers, which are ternary structures with the general formula M_n+1_AX_n_ [24]. The metallic constituents of MXenes typically include early transition metals like Ti, V, and Nb, paired with group 13 and 14 elements such as Al and Si, along with carbon and nitrogen [25]. After etching out the “A” element using hydrofluoric acid or fluoride-containing chemicals, the process yields the two-dimensional layers of M_n+1_X_n_, which are terminated with surface elements classified as T_x_, leading to the generalized formula M_n+1_X_n_T_x_ [26]. Among the various MXenes, niobium carbide-based MXenes (Nb_2_CT_x_) are particularly promising for gas sensing applications, due to their unique physical and chemical properties [27]. Nb_2_CT_x_ MXene, obtained from the selective etching of the precursor Nb_2_AlC, exhibits remarkable electronic properties, along with a large surface area and multiple active sites for gas absorption [28]. The metallic conductivity of Nb_2_CT_x_ enhances charge transfer dynamics, resulting in improved sensing responsiveness [29]. The effectiveness of gas detection in Nb_2_CT_x_ MXene is influenced by functional groups such as –OH, –F, and –O on its surfaces [30]. Furthermore, its layered 2D structure contributes to its higher sensitivity, providing ample binding points that facilitate gas interactions [31]. Nb_2_CT_x_ MXene-based sensors also operate efficiently at room temperature, reducing power usage [32]. Its detection capabilities are quick, with Nb_2_CT_x_ MXene achieving a rapid adsorption and desorption of gas molecules [33]. Due to its stability under standard conditions, Nb_2_CT_x_ MXene is appropriate for continuous gas detection systems [34]. The preparation of Nb_2_CT_x_ MXene starts with the selective removal of aluminum from the MAX phase precursor Nb_2_AlC [35]. Hydrofluoric acid (HF) etching is the primary method, effectively extracting Al while preserving Nb_2_CT_x_ MXene layers [36]. A combination of LiF and HCl serves as etching salts, generating localized HF in the solution to protect the Nb_2_CT_x_ MXene structure and enhance production efficiency [37]. Additionally, a safer approach called alkaline etching for synthesizing Nb_2_CT_x_ MXene can remove aluminum using various solutions without hydrofluoric acid [38]. After etching, delamination can be achieved through sonication or intercalation, yielding monolayer or few-layer Nb_2_CT_x_ MXene structures that improve sensitivity [39]. This shows that gas sensors based on Nb_2_CT_x_ MXene possess outstanding detection capabilities for gases like NO_2_, NH_3_, H_2_S, and volatile organic compounds (VOCs) [40]. They offer excellent selectivity, as functionalized Nb_2_CT_x_ MXene surfaces can be tailored for the detection of specific gases. Operating at parts-per-billion (ppb) levels, these sensors are ideal for environmental monitoring applications. Nb_2_CT_x_ MXene sensors provide immediate response times and quick recovery cycles, significantly outperforming conventional MOS sensors [41,42].

Nb_2_CT_x_ MXene is a promising material for environmental monitoring, particularly in detecting pollutants like NO_2_ and SO_2_, which are crucial for air quality assessments [43]. This technology also identifies dangerous gases in chemical plants and refineries, playing an important role in enhancing industrial safety [44]. In the healthcare sector, Nb_2_CT_x_ MXene is being investigated for its potential in breath tests to detect disease biomarkers [45]. Integrating Nb_2_CT_x_ MXene with IoT devices and wearable tech, it can enable real-time gas detection in portable systems [46]. Nb_2_CT_x_ MXene shows exceptional promise for next-generation gas sensors, boasting improved detection capabilities through greater sensitivity and selectivity, alongside reduced energy demands. Its unique electrical properties, easy surface modification, and compatibility with various applications make it an outstanding gas sensor material. Future research should aim at refining the synthesis methods, enhancing the surface modification selectivity, and promoting the commercial use of Nb_2_CT_x_ MXene for the detection of various gases from both outdoor and indoor environments. Ongoing advancements in Nb_2_CT_x_ MXene-based sensors have significantly affected a range of gas sensing applications.

This review paper explores the properties of Nb_2_CT_x_ MXene, known for its impressive electrical attributes, customizable surface characteristics, and excellent mechanical performance. This review paper discusses synthesis methods, including etching and post-modification, aimed at optimizing surface terminations for improved sensing results. It provides a detailed analysis of Nb_2_CT_x_ MXene’s crystal structure, morphological characteristics, and electronic properties, shedding light on its remarkable detection abilities for NH_3_, serotonin, NO_2_, and VOCs. Additionally, this review highlights the role of Nb_2_CT_x_ MXene nanostructures in enhancing its gas sensing performance and addresses the challenges related to stability and scalability.

## 2. Synthesis, Crystal Structure, and Morphology

Recently, there has been immense research interest in Nb_2_CT_x_ MXene because of its unique electronic properties, its capacity for redox reactions, and its wide-ranging potential applications in sensor technologies. Nb_2_CT_x_ MXene’s synthesis begins with the selective etching of the aluminum layer from the Nb_2_AlC MAX phase, then separating nanosheets into individual or a few layers [28]. The choice of etching technique significantly affects both the structural condition and the surface chemical characteristics, as well as the functional capabilities, of the resulting MXene. The standard acid etching process utilizes highly concentrated hydrofluoric acid to dissolve the A-layer (Al), resulting in multilayered Nb_2_CT_x_ MXene structures with surface terminations of –F, –OH, and –O [47]. The presence of –F terminations in Nb_2_CT_x_ MXene is known to reduce electrical conductivity and restrict the presence of chemically active sites, leading to a decreased sensitivity and charge transfer efficiency in gas sensing applications. Furthermore, the excessive fluorination of Nb_2_CT_x_ MXene may adversely affect long-term stability, particularly under ambient exposure conditions. On the other hand, the safety risks associated with using hydrofluoric acid remain severe due to its high toxicity and corrosive properties. The surface functionalization and partial oxidation of HF-etched Nb_2_CT_x_ MXene occur in an uncontrolled way, resulting in detrimental effects on its physicochemical properties. Therefore, developing in situ HF generation systems represents an alternative strategy to address these issues [48]. A LiF/HCl etching system offers improved control and safety during synthesis by generating HF in situ, thereby minimizing the handling of concentrated HF [49]. This method enhances the safety performance in labs, while also facilitating lithium-ion intercalation during etching, thereby supporting downstream delamination processes. Importantly, the LiF/HCl etching method leads to surfaces of Nb_2_CT_x_ MXene with reduced –F and enhanced –OH and –O terminations, which substantially benefit gas sensing applications. The oxygen-rich and hydroxylated surfaces of Nb_2_CT_x_ MXene enhance conductivity and provide stronger adsorption sites for electron-withdrawing gases such as NO_2_, thereby improving its sensitivity, selectivity, and response speed. Sensors fabricated using LiF/HCl-derived Nb_2_CT_x_ MXenes show a reliable performance, exhibiting better signal-to-noise ratios and quicker recovery times than those produced through direct HF etching. The delamination process of etched multilayer Nb_2_CT_x_ MXene typically begins with intercalation using organic molecules, followed by mild agitation to produce sheets with few or single layers. Several agents, including dimethyl sulfoxide (DMSO) and tetrabutylammonium hydroxide (TBAOH), open the interlayer distance by mitigating van der Waals forces [50]. Delaminated Nb_2_CT_x_ MXene flakes maintain stability as aqueous colloids, while exhibiting a large surface area and excellent dispersibility that support various gas sensing applications.

Recently, fluoride-free synthesis methods for MXenes have been integrated with electrochemical etching and molten salt techniques. Electrochemical etching demonstrates potential as an environmentally friendly and controllable technique for dissolving the Al layer in electrolyte solutions [51]. This synthesis method eliminates toxic fluorides and provides superior control over the surface properties when producing MXenes. Interestingly, the defect-rich surfaces of Nb_2_CT_x_ MXene enhance its electronic conductivity and increase the number of active adsorption sites, which is particularly advantageous for the detection of redox-active gases such as NO_2_. Electrochemically etched Nb_2_CT_x_ MXenes deliver ultra-low detection limits, a rapid response and recovery, and excellent repeatability. Molten salt etching is a fluoride-free alternative that operates in ZnCl_2_ or CuCl_2_ at elevated temperatures [52]. The quality of the MXenes, along with the process’s reproducibility, requires further attention, since high-temperature procedures can lead to structural issues and result in residual impurities. The production of Nb_2_CT_x_ MXene requires further improvements to achieve a standardized synthesis of surfaces with specific terminations, high production volumes, and the preservation of material structures. The combination of etching parameters, intercalation agents, and post-treatment methods entirely determines the performance-related physical and chemical specifications applicable to specific uses of MXenes. The scientific community has inadequately studied various etching approaches for Nb-based MXenes, and it lags behind the well-established research on Ti_3_C_2_T_x_ systems. Research focused on developing diverse synthetic methods for Nb_2_CT_x_ MXene has made notable advancements; however, the standardization of procedures and safer, scalable production must be combined with thorough research on the structure–property relationships affected by synthesis parameters. Efforts to improve synthesis procedures must continue, as they will determine the successful commercial deployment of Nb_2_CT_x_ MXene.

**Figure 1:**
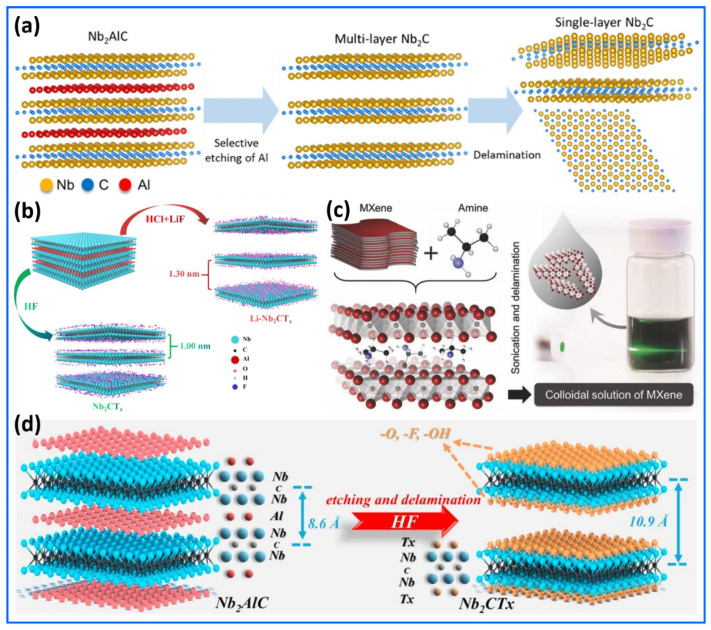
Synthesis methods of Nb_2_CT_x_. (**a**) Schematic illustration of the synthesis process for high-quality single-layer Nb2CT_x_ MXene. The production process begins with the selective removal of aluminum (Al) from Nb2AlC MAX material through concentrated hydrofluoric acid (HF) treatment, followed by the formation of a Nb2CT_x_ MXene structure that appears as accordion-shaped multilayers. The use of tetramethylammonium hydroxide (TMAOH) facilitates the delamination of layers, resulting in the layered separation of single-layer Nb2CT_x_ MXene nanosheets. Reproduced with permission from Ref. [53]. Copyright (2022) American Chemical Society. (**b**) The synthesis of Li-preintercalated Nb_2_CT_x_ (Li-Nb_2_CT_x_) MXene from Nb_2_AlC occurs in a single step using a combined HCl/LiF solution. This synthesis method involves the selective etching of Al layers, which introduces lithium ions during the process, creating an expanded interlayer space while producing –O/–OH surface group terminations. Reproduced with permission from Ref. [54]. Copyright (2021) Elsevier. (**c**) Schematic representation of the amine-assisted delamination process for Nb_2_CT_x_ MXene. The two-step procedure involves the initial intercalation of isopropylamine (i-PrA) molecules between the Nb_2_CT_x_ layers, followed by mild sonication in de-aerated, deionized water to yield few-layered or single-layer flakes. The molecular model depicts Nb (red), C (black), N (blue), and H (white) atoms; surface terminations are omitted for clarity. The photograph on the right demonstrates the Tyndall effect in the resulting colloidal dispersion, confirming the formation of a stable suspension of exfoliated Nb_2_CT_x_ flakes. Reproduced with permission from Ref. [55]. Copyright (2015) Wiley-VCH GmbH. (**d**) Schematic illustration of the synthesis process of Nb_2_CT_x_ MXene from the Nb_2_AlC MAX phase. The two-step etching procedure selectively removes Al atoms using hydrofluoric acid (HF). Reproduced with permission from Ref. [56]. Copyright (2021) Elsevier.

The synthesis methods of Nb_2_CT_x_ are presented and discussed in Figure 1a–d [53,54,55,56]. The synthesis of high-quality single-layer Nb_2_CT_x_ is presented with a structured synthesis methodology, as illustrated in Figure 1a [53]. The first step of their synthesis, illustrated in Figure 1a, involves following the MAX-phase Nb_2_AlC through the selective removal of aluminum atoms using concentrated hydrofluoric acid (HF). A chemical treatment solution removes the aluminum atomic layers from Nb_2_AlC materials, transforming the material into accordion-shaped Nb_2_CT_x_ MXene. The single-layer delamination process for multilayered MXene takes place when tetramethylammonium hydroxide (TMAOH) is applied between the layers to facilitate exfoliation. TMAOH exposure yields Nb_2_CT_x_ nanosheets composed of single atomic layers, accompanied by a significant increase in interlayer spacing (d-spacing). A schematic diagram in Figure 1a illustrates that selecting the etching of Al and chemical delamination methods as key elements of structural engineering facilitates the optimization of Nb_2_CT_x_ MXene for diverse applications. Moreover, a one-step process for creating Li-preintercalated Nb_2_CT_x_ (Li-Nb_2_CT_x_) MXene is interestingly illustrated in the schematic depiction shown in Figure 1b [54]. A different method for producing Li-Nb_2_CT_x_ MXene is used, moving away from the traditional HF-based etching of MAX phases. This new approach employs a combination of hydrochloric acid (HCl) and lithium fluoride (LiF). Replacing HF enhances safety, simplifies reaction processes, and produces positive chemical and structural effects. Furthermore, Figure 1b illustrates the simultaneous etching of Al layers from Nb_2_AlC and the preintercalation of Li^+^ ions into the Li-Nb_2_CT_x_ MXene. This dual-action mechanism offers threefold benefits: (i) it increases the interlayer spacing due to the co-intercalation of Li^+^ and water molecules, enhancing ion diffusion kinetics; (ii) it reduces the presence of –F functional groups while increasing –O and –OH terminations, which are more electrochemically active for Li+ storage; and (iii) it preserves the crystalline quality of the Li-Nb_2_CT_x_ MXene due to the mild reaction conditions. Figure 1c provides a clear and informative schematic of the two-step delamination process for Nb_2_CT_x_ MXene, using isopropylamine (i-PrA) as an intercalant [55]. This method represents a significant advancement in MXene processing, especially for niobium-based members of the family, which have previously shown resistance to delamination by conventional agents like dimethyl sulfoxide (DMSO). Intercalation represents the initial stage in which Nb_2_CT_x_ multilayers absorb aqueous i-PrA. The etched Nb_2_CT_x_ MXene surfaces display acidic properties, featuring hydroxyl groups that facilitate the intercalation of protonated amine species (R-NH_3_^+^) through electrostatic attraction between the layers. i-PrA shows advantageous penetration characteristics due to its small size and moderate hydrophobicity, allowing it to easily enter the interlayer space and effectively separate the layers by reducing van der Waals forces. The image in Figure 1c showing the Tyndall effect serves as clear experimental proof of successful exfoliation. The laser beam passing through the Nb_2_CT_x_ flake colloidal suspension illustrates the stable existence of nanoscale particles, indicating a stable dispersion. Therefore, it is concluded that Figure 1c encapsulates a crucial methodological advance in Nb_2_CT_x_ MXene chemistry by illustrating a non-destructive, amine-mediated delamination strategy for Nb_2_CT_x_. The synthesis strategy for Nb_2_CT_x_ MXene, as illustrated in Figure 1d, represents a significant advancement in the processing of Nb_2_CT_x_ MXene [56]. This two-step chemical etching process begins with the selective removal of aluminum layers from the Nb_2_AlC MAX phase using hydrofluoric acid (HF). The etching preferentially disrupts the weaker Nb–Al bonds over the stronger Nb–C bonds, resulting in the exfoliation of the layered structure. Subsequently, the newly exposed surfaces of Nb_2_C interact with water and fluoride ions to form a combination of surface terminations, including –F, –OH, and =O groups. The structural transformation from a dense MAX phase to a loosely stacked, lamellar Nb_2_CT_x_ MXene introduces a substantial increase in interlayer spacing (~10.9 Å).

XRD is essential for phase identification, structure analysis, and evaluations of interlayer distances in Nb-based MXenes. X-ray diffraction (XRD) spectra provide crucial evidence regarding developing the Nb_2_CT_x_ MXene crystal structure as the Nb_2_AlC MAX phase transforms. The removal of Al atomic layers and the formation of two-dimensional (2D) lamellar structures result in significant changes to the diffraction pattern, accompanied by surface termination bonds known as T_x_, which include –OH, –F, and –O. The synthesized pristine Nb_2_AlC MAX phase exhibits distinct, clear peaks in its XRD pattern, indicating a perfectly ordered hexagonal crystal structure. The XRD pattern of Nb_2_AlC MAX displays an intense and dominant lattice plane (104) at 2θ = 39°, indicating that the layered framework retains Nb, Al, and C atoms [57]. The (104) lattice plane in XRD patterns disappears once the Al layers are selectively removed from the Nb_2_AlC MAX using hydrofluoric acid (HF) or generates HF in situ through the reaction between LiF and HCl. The changes in the low-angle region become notable as a strong (002) reflection develops between 2θ values of 6° and 9° in Nb_2_CT_x_ MXene, depending on the extent of surface modifications and interlayer insertions [56]. The (002) reflection in XRD results in an expanded interlayer spacing (d-spacing) observed between 12 Å and over 20 Å due to the presence of terminal functional groups, water molecules, or organic ions within the Nb_2_CT_x_ MXene [58]. The extent of peak shift indicates the degree to which chemical intervention and the presence of water have modified the structure of the Nb_2_CT_x_ MXene. When multilayer Nb_2_CT_x_ MXene sheets undergo mechanical or acoustic thinning into single or few layers, the (002) peak exhibits broadening features alongside a reduction in XRD peak intensity. This effect is associated with decreased stack ordering and a reduction in c-axis crystallite thickness in Nb_2_CT_x_ MXene. The ultrathin 2D flakes, coupled with the loss of long-range order, result in the significant suppression or disappearance of the reflections (004) and (006) in highly exfoliated Nb_2_CT_x_ MXenes [59]. Changes in the XRD spectra that track the disappearance of the (104) peak and the development and movement of the (002) peak provide distinctive proof regarding the conditions necessary for Nb_2_CT_x_ MXene’s synthesis and delamination.

The Raman spectrum of Nb_2_CT_x_ MXene provides a distinct identification fingerprint that reveals both the transformation from MAX phase to MXene and the impact of terminal groups on the structure and disorder. Furthermore, Raman spectroscopy is a vital analytical technology for elucidating the structural and vibrational properties and chemical interactions of two-dimensional (2D) MXenes. The Nb_2_CT_x_ MXene, which results from stripping the aluminum layer out of the Nb_2_AlC parent MAX phase, exhibits a Raman pattern that illustrates how exfoliation processes, surface termination methods, and structural irregularities alter its vibrational modes. The Raman spectra of Nb_2_CT_x_ MXene exhibit various vibrational bands spanning from low to mid frequencies, as the vibrations of niobium and carbon atoms interact with the 2D structure. The Nb atomic in-plane and out-of-plane vibrations in Nb_2_CT_x_ MXene give rise to vibrational modes within 100–300 cm^−1^ [60]. The deformations in the Nb–C lattice create vibrational modes that help validate the layer architecture after etching. The Raman-active modes located between 300 and 600 cm^−1^ represent Nb–C bond stretching motions, as well as bending vibrations [61]. The sensitivity of these features arises from various surface terminations (T_x_) introduced through etching and delamination methods, including –OH, –F, and =O groups. Surface terminal groups with varying electronegativity values and atomic masses induce substantial changes in bond force constants, resulting in fluctuations in peak intensities and positions. Raman spectroscopy has significant potential as an evaluation method for both surface chemistry and modifications to post-synthesis Nb_2_CT_x_ MXene surfaces. The 600–800 cm^−1^ spectral range displays vibrational bands that likely originate from Nb–O and C–O vibrations, suggesting either partial oxidation or the presence of oxygen-terminal groups on the surface of Nb_2_CT_x_ MXene [62]. Beyond 800 cm^−1^, Nb_2_CT_x_ MXene exhibits weak Raman activity, along with potentially observed broad bands that similarly correspond to the D (~1350 cm^−1^) and G (~1580 cm^−1^) peaks of carbon, mainly when residual graphitic carbon is present [63]. In addition, the Raman spectrum of Nb_2_CT_x_ MXene exhibits significantly broadened and less symmetrical features, which differ substantially from those of its precursor, the MAX phase Nb_2_AlC. The structural modifications of the MAX-phase Nb_2_AlC result from both the removal of Al layers and the introduction of surface functional groups, accompanied by an increase in structural disorder. After etching, Nb_2_CT_x_ MXene exhibits broad and downshifted peaks, indicating both successful exfoliation and changes in the chemical structure of Nb and C atoms. Interestingly, Raman spectroscopy enables the real-time observation of oxidative progression and surface termination homogeneity, as well as delamination quality, without damaging the Nb_2_CT_x_ MXene [64]. Moreover, Raman spectroscopy capitalizes on changes in Nb–C modes and the appearance of new oxygen-related vibrational modes. Raman spectroscopy yields an infrared signature of Nb_2_CT_x_ MXene that reveals three critical aspects, encompassing its origin from the MAX phase and changes resulting from surface coverage and crystal disordering [65].

The surface chemistry and elemental composition of transition metal carbides and nitrides fall into the two-dimensional category, which can be analyzed using critical X-ray photoelectron spectroscopy (XPS). XPS provides essential information about the evolution of niobium-based MXene (Nb_2_CT_x_) from its parent MAX phase (Nb_2_AlC), including its surface termination characteristics and chemical environments, as these factors influence its physical and chemical responses [66]. The survey spectrum of Nb_2_CT_x_ MXene shows strong signals from Nb, C, O, and F but exhibits a complete absence of Al peaks, indicating that selective etching has effectively removed the Al layers [67]. This transformation confirms the successful synthesis of the Nb_2_CT_x_ MXene phase from the Nb_2_AlC precursor. A high-resolution scan of the Nb 3d region of Nb_2_CT_x_ MXene typically reveals complex identifications of multiplet patterns due to spin–orbit coupling and several overlapping oxidation states [68]. The two peaks at 203.0 eV (Nb 3d_5/2_) and 206.0 eV (Nb 3d_3/2_) signify the formation of Nb–C bonds, demonstrating that the carbide network remains intact within the Nb_2_CT_x_ MXene structure [69]. The doublet peaks between 207 and 210 eV suggest the presence of oxidized Nb species, such as Nb^4+^ and Nb^5+^, indicating surface oxidation or the presence of terminal oxygen or hydroxyl groups (–O, –OH) [70]. Some peaks at 206–207 eV suggest Nb–F bonding, likely due to the conditions created by hydrofluoric acid during etching procedures [71]. In the Nb_2_CT_x_ MXene XPS spectra, the C 1s peak displays three distinct peaks, one at 282.0 eV attributed to Nb–C bonds, 284.5 eV representing adventitious C=C and C–C hybridization, and higher binding energy peaks ranging from 286.0 to 288.0 eV, indicating the presence of C–O and C=O functional groups [72]. The Nb–C signal intensity, compared to other carbon signals, suggests an indirect relationship that reflects the preserved structure of the Nb_2_CT_x_ MXene. The O 1s spectra undergo deconvolution to uncover multiple oxygen-containing surface terminations present on Nb_2_CT_x_ MXene surfaces [73]. Additionally, Nb–O bonds corresponding to the peaks at 530.0 eV are due to the partial oxidation of surface Nb atoms of the Nb_2_CT_x_ MXene [74]. XPS peaks at 531.0 eV typically indicate water adsorption or physisorbed –OH groups of the Nb_2_CT_x_ MXene [75]. The high amount of oxygen terminations in Nb_2_CT_x_ MXene results in greater water affinity and enhanced stability, which supports processing and functionalization efforts. The excessive fluorination of Nb_2_CT_x_ MXene surfaces hinders electronic conductivity; however, fluorine termination enhances stability. The –F functionalization on the Nb_2_CT_x_ MXene surface results in the appearance of the F 1s peak at 685 eV, while the presence of –OH and =O groups contributes to an increased intensity of the O 1s peak [56]. Additionally, the Nb 3d_3/2_ and 3d_5/2_ peaks observed in the XPS spectra of NbC_x_O_y_ originate from surface terminations involving –O and –OH groups, as well as from adsorbed water molecules, which shows the transitions in the surface termination chemistry, suggesting changes in the nature or composition of the functional groups present on the surface [76]. The combination of XPS-analyzed termination chemistry and ratios determines the properties of the Nb_2_CT_x_ MXene, influencing its surface energy behavior, interlayer distance, and redox capabilities. The XPS results of the Nb_2_CT_x_ MXene reveal that the structural features of carbides are due to changes in surface behavior. Furthermore, post-etching analysis shows that the carbide core structure remains mostly intact. Simultaneously, surface oxidation and functionalization occur inevitably, offering numerous benefits [77].

**Figure 2:**
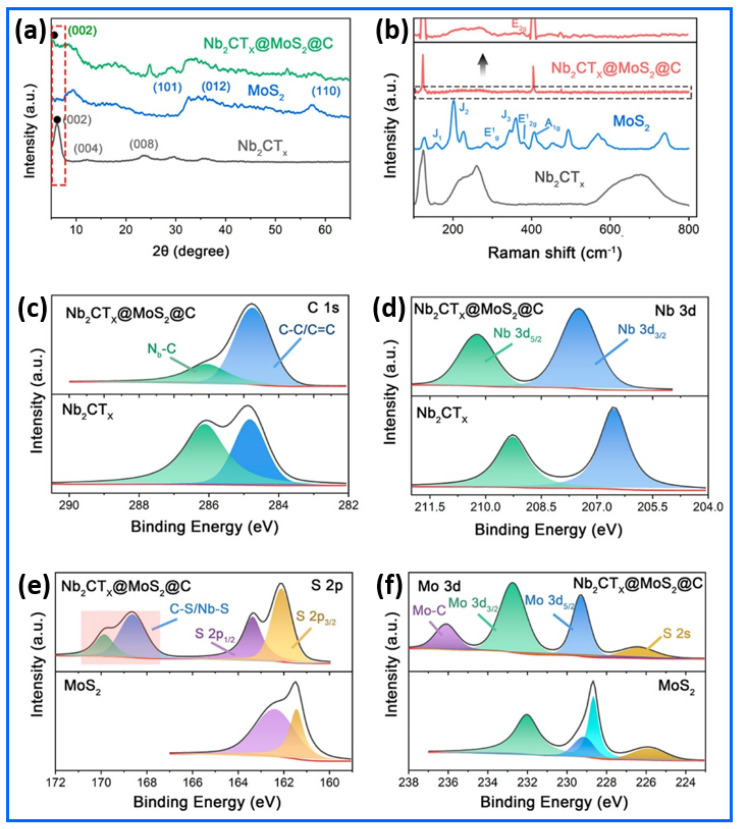
Structural and compositional characterizations of the Nb_2_CT_x_@MoS_2_@C hybrid. (**a**) X-ray diffraction (XRD) patterns and (**b**) Raman spectra of Nb_2_CT_x_@MoS_2_@C, MoS_2_, and Nb_2_CT_x_, confirming successful hybrid formation and a distinct phase composition. X-ray photoelectron spectroscopy (XPS) spectra of the Nb_2_CT_x_@MoS_2_@C hybrid. (**c**) C 1s spectrum indicating a carbonaceous element, (**d**) Nb 3d spectrum verifying the presence and oxidation state of niobium, (**e**) S 2p spectrum confirming the incorporation of MoS_2_, and (**f**) Mo 3d spectrum showing the chemical states of molybdenum. Reproduced with permission from Ref. [78]. Copyright (2021) American Chemical Society.

The structural and chemical characteristics of the Nb_2_CT_x_@MoS_2_@C hybrid were comprehensively elucidated through X-ray diffraction (XRD), Raman spectroscopy, and X-ray photoelectron spectroscopy (XPS), as illustrated in Figure 2a–f [78]. The X-ray diffraction (XRD) analysis in Figure 2a confirms that the synthesized Nb_2_CT_x_@MoS_2_@C hybrid structure was successfully synthesized. The (002) XRD peak of Nb_2_CT_x_ MXene shifted from its original position at 13.09° to approximately 8.92°, indicating that the aluminum layers were successfully removed through etching, resulting in expanded, few-layered Nb_2_CT_x_ nanostructures. The (002) peak in the Nb_2_CT_x_@MoS_2_@C hybrid exhibits a new position at 5.20°, due to the additional expansion of interlayer distances induced by MoS_2_ and carbon additives. The expansion directly benefits sodium-ion intercalation by creating more efficient transport paths and enhancing the volume stability throughout electrochemical charging. The in situ growth of MoS_2_ nanosheets on the MXene framework is evident from the emergence of the (002) and (101) planes at 14.5° and 33°, respectively. The presence of these characteristic peaks confirms the formation of the Nb_2_CT_x_@MoS_2_@C hybrid structure, as no Nb_2_AlC MAX-phase peaks are detected at 38.93°. Additional information about the phase composition and structural integration of the Nb_2_CT_x_@MoS_2_@C hybrid can be obtained using Raman spectroscopy. According to its distinctive vibrations, the Nb_2_CT_x_ MXene exhibits broad characteristics at 132, 200–300, and 550–750 cm^−1^, as shown in Figure 2b. In addition to further signals at 156, 206, and 346 cm^−1^, which are associated with the J_1_, J_2_, and J_3_ modes of the metallic 1T phase, MoS_2_ exhibits peaks at ~283 cm⁻^1^ (E_1_g of 1T-MoS_2_), 383 cm⁻^1^ (E_2_g^1^ of 2H-MoS_2_), and 404 cm⁻^1^ (A_1_g of 2H-MoS_2_). Interestingly, the prominence of the 383 cm^−1^ (E_2g_^1^) peak in the Nb_2_CT_x_@MoS_2_@C hybrid indicates the preferential stability of the semiconducting 2H-MoS_2_ phase. The XPS C 1s spectrum of the Nb_2_CT_x_@MoS_2_@C hybrid in Figure 2c reveals key insights into the surface chemistry and bonding states of carbon within the Nb_2_CT_x_@MoS_2_@C hybrid. The peaks centered at 284.7 eV and 286.2 eV correspond to graphitic C=C/C–C and C–O species, respectively. Notably, the position and intensity of these peaks remain relatively unchanged before and after the integration of MoS_2_, indicating that the carbon coating derived from polydopamine (PDA) remains chemically stable during the Nb_2_CT_x_@MoS_2_@C hybrid’s formation. This stable carbon layer plays a crucial role in enhancing the electronic conductivity and structural integrity of the Nb_2_CT_x_@MoS_2_@C hybrid. Figure 2d presents the Nb 3d spectra, highlighting the electronic interaction between Nb_2_CT_x_ and MoS_2_ in the Nb_2_CT_x_@MoS_2_@C hybrid. In pristine Nb_2_CT_x_, the Nb 3d_5/2_ and 3d_3/2_ peaks appear at approximately 206.6 eV and 209.2 eV, respectively. After MoS_2_’s integration, these peaks shift to 207.4 eV and 210.3 eV, indicating a redistribution of electron density around the Nb centers. This shift reflects the formation of interfacial bonds between Nb and MoS_2_, suggesting a strong chemical interaction that facilitates an efficient charge transfer across the Nb_2_CT_x_@MoS_2_@C heterojunction interface. The S 2p region shown in Figure 2e further confirms the integration of MoS_2_ into the Nb_2_CT_x_. Peaks at 161.5 eV and 162.2 eV correspond to S 2p_3/2_ and S 2p_1/2_, respectively, indicative of sulfide (S^2−^) states typical of MoS_2_. Additional features with higher binding energies, around 168.7 and 170.1 eV, suggest the presence of oxidized sulfur species or interfacial bonding (Nb–S and C–S). In Figure 2f, the Mo 3d spectra illustrate the oxidation states of molybdenum in the Nb_2_CT_x_@MoS_2_@C hybrid. The Mo 3d_5/2_ and 3d_3/2_ peaks at 229.5 and 232.7 eV, respectively, align with Mo^4+^ in MoS_2_. Minor shifts in binding energy and the emergence of a weak peak around 236.2 eV may suggest Mo–C bonding or a slight surface oxidation in the Nb_2_CT_x_@MoS_2_@C hybrid. Additionally, the maintenance of the Mo^4+^ oxidation state, even after carbonization, indicates that the structural integrity of MoS_2_ remains intact.

**Figure 3:**
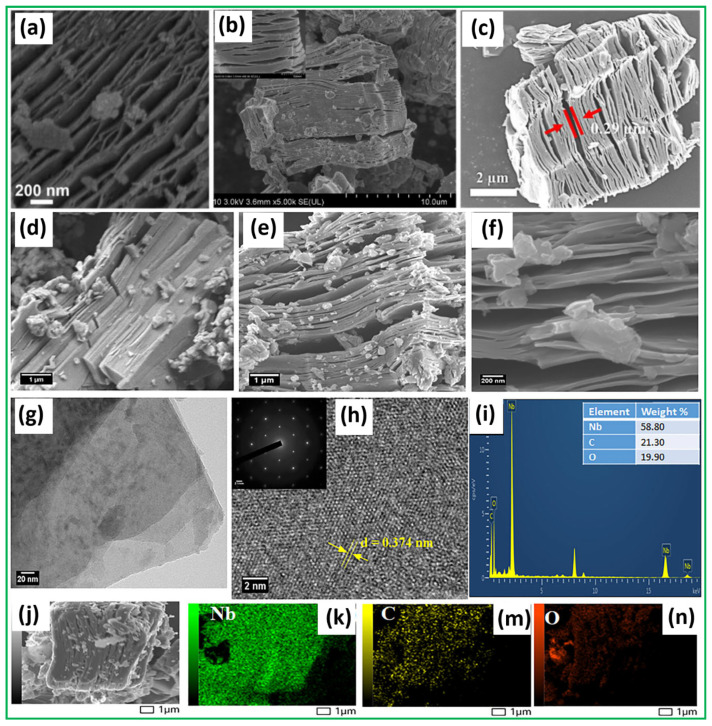
(**a**) The scanning electron microscopy (SEM) image of Nb_2_CT_x_ MXene depicts a characteristic accordion-like, multilayered structure that forms after the selective etching of Nb_2_AlC. This morphology, resulting from the removal of Al layers, provides an expanded interlayer spacing, enhances the surface area, and facilitates an efficient charge transfer. The layered configuration is crucial for its function as a conductive substrate and cocatalyst in the hierarchical CdS@Nb_2_O_5_/Nb_2_CT_x_ heterojunction. Reproduced with permission from Ref. [79]. Copyright (2023) American Chemical Society. (**b**) SEM image of Nb_2_CT_x_ MXene after the selective Al etching process on the Nb_2_AlC MAX phase. The inset image of a block section reveals more extensive details, demonstrating the essential separation and looseness of Nb_2_CT_x_ MXene layers, which facilitate the effective intercalation of alkali ions. Reproduced with permission from Ref. [80]. Copyright (2021) American Chemical Society. (**c**) SEM image of Nb_2_CT_x_ MXene obtained after HF etching of the parent Nb_2_AlC MAX phase. The partially interconnected lamellae suggest the presence of structural defects or a non-uniform Al distribution. Reproduced with permission from Ref. [81]. Copyright (2020) Elsevier. (**d**) FESEM image of Nb_2_AlC showing the compact layered structure of the MAX phase. (**e**,**f**) FESEM images of Nb_2_CT_x_ illustrating the expanded, exfoliated layered morphology following Al removal. (**g**) HRTEM image of few-layered Nb_2_CT_x_ nanosheets. (**h**) High-resolution TEM image of Nb_2_CT_x_ with an interplanar spacing of 0.374 nm (inset shows the SAED pattern with sixfold symmetry, confirming crystalline order). (**i**) EDX spectrum of Nb_2_CT_x_ MXene confirming the presence of Nb, C, and O. (**j**) Selected area of Nb_2_CT_x_ FESEM image and corresponding elemental mapping images for Nb (**k**), C (**m**), and O (**n**) demonstrating a uniform elemental distribution across the Nb_2_CT_x_ MXene. Reproduced with permission from Ref. [82]. Copyright (2024) Wiley-VCH GmbH.

Figure 3a shows that Nb_2_CT_x_ MXene has an accordion-shaped multilayer structure, displaying the characteristic MXene morphology formed during etching procedures [79]. The sequence of aluminum layer removal from the MAX-phase Nb_2_AlC creates hierarchical structures that enhance both the spacing between layers and surface availability. The intrinsic metallic character of Nb_2_CT_x_ MXene enables fast interband electron transport, which is necessary to reduce charge recombination events. The structural advantage lies in its ability to ensure the proper development of the CdS@Nb_2_O_5_/Nb_2_CT_x_ hierarchical heterojunction. Figure 3b presents a SEM image, which clearly and inspiringly depicts the surface morphology of the synthesized Nb_2_CT_x_ MXene [80]. The SEM image shows an accordion-shaped layered arrangement that develops during the selective removal of Al layers from the MAX-phase precursor (Nb_2_AlC) to produce well-exfoliated MXene materials. The SEM image, particularly its magnified inset, highlights the significant degree of interlayer separation among the Nb_2_CT_x_ MXene sheets. The mechanical agitation, combined with delamination processes, enhances the gaps between the layers that result after Al’s removal through etching. The SEM image of the Nb_2_CT_x_ MXene reveals a textured and slightly rough surface topology, indicating structural defects and vacancies that enhance its surface properties for various applications. Furthermore, the SEM image in Figure 3c illustrates the result of removing the Al layer from the Nb_2_AlC MAX phase using hydrofluoric acid etching to obtain Nb_2_CT_x_ MXene [81]. The developed morphology shows accordion-like layers that parallel the characteristic structure of well-etched MXene materials. The structural transformation indicates the complete success of the etching procedure, because it led to the removal of Al atoms and the revelation of two-dimensional Nb_2_CT_x_ MXene nanosheets. The SEM image demonstrates that the present Nb_2_CT_x_ MXene layers do not achieve full delamination but preserve some interconnecting properties. Two possible explanations for this observation are the non-homogeneous Al phase distribution in the Nb_2_AlC structure and natural defects found in the MAX structure. Nb_2_CT_x_ MXene exhibits an ideal interlayer structure for the in situ development and insertion of Nb_2_O_5_ nanoparticles. The built-in interlayer space of the Nb_2_CT_x_ MXene structure enables additional expansion during hybridization, so it accepts more electromagnetic waves and produces better scattering results and a reduced reflection. In addition, the structure of Nb_2_CT_x_ MXene, along with its elemental constituents, was thoroughly investigated using a combination of FESEM, HRTEM, SAED, EDX, and elemental mapping techniques, as highlighted in Figure 3d–k,m,n [82]. The FESEM micrograph in Figure 3d reveals a tightly packed layered morphology for the pristine Nb_2_AlC MAX phase, consistent with its known crystalline structure. Following the etching and delamination process, Figure 3e,f displays a significant expansion of the interlayer distance, accompanied by a crumpled, accordion-shaped structure in Nb_2_CT_x_ MXene. The selective HF treatment results in morphological changes, because it removes the Al atomic layer. The expanded structure creates a larger accessible surface area and meso/macroporous domains crucial for efficient ion movement in supercapacitors. An HRTEM image provided in Figure 3g verifies the production of ultrathin Nb_2_CT_x_ MXene flakes. Figure 3h displays clear lattice lines with an interplanar distance of 0.374 nanometers, corresponding to the (002) lattice plane. The SAED pattern [inset of Figure 3h] exhibits a distinct sixfold symmetry, indicating the crystalline nature of the Nb_2_CT_x_ MXene basal plane. The EDX spectrum of Figure 3i reveals niobium (Nb), carbon (C), and oxygen (O) as elemental constituents, with relative weight percentages of 58.8%, 21.3%, and 19.9%, respectively. Figure 3j,k,m,n shows the FESEM image and corresponding elemental mapping, which reveal a uniform distribution of Nb, C, and O elements, confirming the homogeneity in Nb_2_CT_x_ MXene.

## 3. Properties

### 3.1. Optical

Understanding the optical behavior of Nb_2_CT_x_ MXene and that at the interface of its heterostructures is critical for optimizing charge carrier dynamics in advanced optoelectronic and photocatalytic systems. In this context, the introduction of Nb_2_CT_x_ MXene as a functional interfacial layer in hybrid structures has demonstrated promising outcomes. Specifically, the steady-state photoluminescence (PL) and time-resolved PL (TRPL) analyses offer essential insights into charge dynamics at the perovskite/Nb_2_CT_x_ interface [83]. The introduction of the Nb_2_CT_x_ layer results in a notable decrease in PL intensity, which indicates effective hole extraction and a reduction in radiative recombination losses. TRPL spectra show quicker decay dynamics in films with the interface, confirming improved charge separation. Therefore, the Nb_2_CT_x_ interface is crucial for enhancing carrier dynamics, enabling efficient hole transport, and reducing energy loss pathways, ultimately boosting the device’s performance and operational stability. This trend is also evident in more complex nanocomposite architectures. For instance, in the Nb_2_CT_x_/UiO-66@rGQDs nanocomposite architecture, the presence of Nb_2_CT_x_ leads to a further reduction in PL emissions compared to pristine UiO-66 and rGQDs-modified UiO-66, indicating an improved suppression of electron–hole recombination [84]. Integrating rGQDs with UiO-66 markedly lowers PL intensity relative to the unmodified UiO-66 and rGQDs, indicating a decrease in electron–hole recombination. The addition of Nb_2_CT_x_ further diminishes PL emissions, highlighting its role in enhancing charge separation and improving photocatalytic efficiency. Furthermore, the absorption edge shows a red shift from 306 nm for UiO-66@rGQDs to 395 nm for Nb_2_CT_x_/UiO-66@rGQDs, indicating an improved capability for light harvesting. These findings collectively highlight the strategic role of Nb_2_CT_x_ in modulating interfacial optical properties, facilitating efficient charge dynamics, and enhancing the overall device performance.

**Figure 4:**
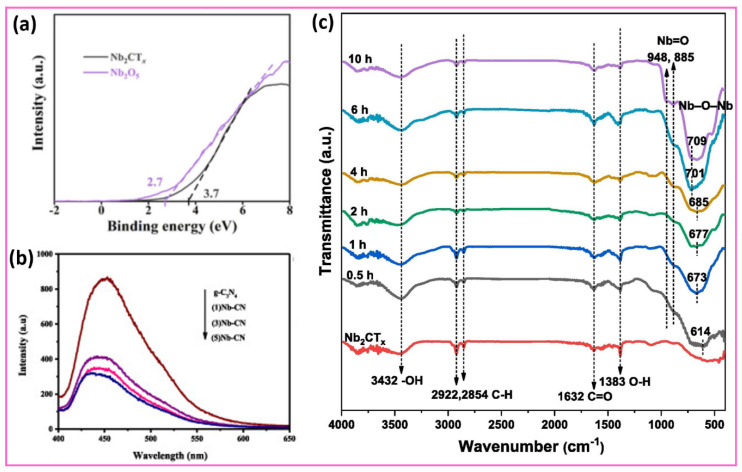
(**a**) Ultraviolet photoelectron spectroscopy (UPS) valence-band spectra of Nb_2_CT_x_ MXene and Nb_2_O_5_, showing VBM values of 3.7 eV and 2.7 eV, respectively. Reproduced with permission from Ref. [85]. Copyright (2023) Elsevier. (**b**) Photoluminescence (PL) spectra of pristine g-C_3_N_4_ (CN) and its composites with varying loadings of Nb_2_CT_x_ MXene (1 wt%, 3 wt%, and 5 wt%), denoted as (1)Nb–CN, (3)Nb–CN, and (5)Nb–CN, respectively. Reproduced with permission from Ref. [86]. Copyright (2023) Elsevier. (**c**) FTIR spectra analysis of Nb_2_CT_x_ and Nb_2_O_5_@Nb_2_CT_x_, revealing the changes in surface functional groups during the hierarchical heterostructure’s formation. Reproduced with permission from Ref. [87]. Copyright (2021) Elsevier.

To further clarify the underlying mechanisms of these improved charge transfer processes, studying the electronic band structure is essential. In this regard, ultraviolet photoelectron spectroscopy (UPS) provides critical insights into the energetic alignment at the interface. The valence band (VB) spectra of Nb_2_CT_x_ MXene and Nb_2_O_5_ are estimated using ultraviolet photoelectron spectroscopy (UPS), as depicted in Figure 4a [85]. The energy-level alignment of the Nb_2_CT_x_/Nb_2_O_5_ Schottky heterostructure interface is crucial for understanding its electrochemical properties, as it determines the alignment of energy levels. The VBM energy level of Nb_2_CT_x_ MXene reaches 3.7 eV, compared to the VBM energy position of Nb_2_O_5_, which is at 2.7 eV. The valence band of Nb_2_O_5_ exhibits an energy position that is higher than that of Nb_2_CT_x_ MXene. Figure 4b illustrates the photoluminescence (PL) spectra of the g-C_3_N_4_ (CN) and Nb_2_CT_x_/g-C_3_N_4_ (Nb–CN) heterojunctions, such as (1)Nb–CN, (3)Nb–CN, and (5)Nb–CN heterojunctions [86]. Photons are generated from a pristine g-C_3_N_4_ (CN) emission at a high intensity, yet this intensity decreases in all Nb_2_CT_x_/g-C_3_N_4_ (Nb–CN) heterojunctions. The integration of Nb_2_CT_x_ with g-C_3_N_4_ through the (1)Nb–CN, (3)Nb–CN, and (5)Nb–CN heterojunction formation results in a significant reduction in photoluminescence emission intensity. PL intensity decreases because g-C_3_N_4_ forms a well-functioning Schottky heterojunction at its junction with Nb_2_CT_x_ MXene. Photogenerated electrons and holes separate efficiently, because electrons become captured at the Nb_2_CT_x_ MXene’s surface through the Schottky barrier, thus blocking their radiative recombination. The photocatalytic activity becomes more efficient when the (3)Nb–CN and (5)Nb–CN heterojunctions exhibit minimal photoluminescence, owing to their excellent electron–hole separation capabilities. Figure 4c shows the Fourier-Transform Infrared (FTIR) spectra, which provide essential information about surface functional groups and the chemical interactions occurring during the formation process of the Nb_2_O_5_@Nb_2_CT_x_ heterostructure [87]. Surface chemistry is crucial for various applications, as it demonstrates and transforms –OH, C–H, and Nb–O bands with increasing hydrothermal treatment times. The bands at 3432 cm^−1^ and 1383 cm^−1^ in Nb_2_CT_x_ correspond to –OH stretching and bending vibrations, respectively, from hydroxyl terminations. These signals indicate that hydroxyl terminations exist in significant amounts, as they lower the work function of the Nb_2_O_5_@Nb_2_CT_x_ heterostructure to 2.7 eV, while facilitating hole retention. The presence of –OH-terminated MXenes aligns with previous theoretical expectations and experimental research, which show their effectiveness as photocatalytic hole mediators in water decomposition reactions. The surface also contains carbonaceous species alongside partially oxidized structures, as indicated by the weak C–H stretching bands at 2922 cm^−1^ and 2854 cm^−1^, along with the C=O vibration at 1632 cm^−1^. The hydrothermal reaction time influences the formation of Nb_2_O_5_ crystallites, reflecting structural changes and oxidation of Nb_2_CT_x_. The development of Nb–O and Nb–O–Nb bonds becomes apparent as low-wavenumber bands emerge between 700 and 600 cm^−1^, and peaks at 948 cm^−1^ and 885 cm^−1^ demonstrate the successful synthesis of Nb_2_O_5_. The 614 cm^−1^ band exhibits both a blue shift and increased intensity along with the reaction time, indicating the formation of a robust chemical bond and structural integration between the forming Nb_2_O_5_ and the Nb_2_CT_x_ at their interface. The spectroscopic changes offer vital insights into interfacial charge behavior. The conversion from 2D Nb_2_CT_x_ to 1D Nb_2_O_5_ nanorods facilitates an ordered charge separation system, as MXenes store holes while Nb_2_O_5_ exhibits high photoactivity. It is concluded that the anisotropic Nb_2_O_5_ and hydroxyl-terminated MXenes exhibit superior interfacial charge kinetics, based on strong evidence, further affirming this method as a leading approach for developing advanced photocatalytic materials.

### 3.2. Optoelectronic

Nb_2_C exhibits metallic properties; however, certain surface terminations can induce a minor bandgap or modify states near the Fermi level, resulting in semi-metallic and semiconducting characteristics [88]. Nb_2_CT_x_ MXene provides tunability in its band structure, making it appealing for optoelectronic devices used in switching, field-effect transistors, and photodetectors [89]. Nb_2_CT_x_ MXene demonstrates an outstanding capability to absorb light across the full ultraviolet (UV) to near-infrared (NIR) spectrum [90]. These broad absorption characteristics stem from interband transitions and plasmonic effects from niobium d-electrons. Nb_2_CT_x_ MXene’s strong photothermal response is linked to its significant light–matter interactions, which are advantageous for photothermal imaging, therapeutic applications, and solar-powered thermal devices [91]. Time-resolved spectroscopy illustrates that Nb_2_CT_x_ MXene exhibits swift carrier kinetics, which enhances its high-speed optoelectronic and photonic performance [92]. The localized surface plasmon resonance of Nb_2_CT_x_ MXene, along with its broad absorption, is most pronounced in the NIR spectrum [93]. These superior electrical properties, paired with the optical features of Nb_2_CT_x_ MXene, make it a prime material for creating photodetectors and energy harvesting systems. Nb_2_CT_x_ MXene’s performance in energy devices is enhanced when combined with perovskites, organic semiconductors, or quantum dots in heterostructures, improving charge transport and interfacial engineering processes [94]. The advantages of Nb_2_CT_x_ MXene for flexible and wearable optoelectronic devices are attributed to its processability in solution, mechanical flexibility, and high conductivity. Thin Nb_2_CT_x_ MXene films are effective as transparent conducting electrodes in solar cells, as well as in displays and touchscreens [95]. Its inherent metallic nature, combined with its adjustable electronic and optical properties, makes Nb_2_CT_x_ MXene an excellent material for the future development of photonic and optoelectronic devices.

**Figure 5:**
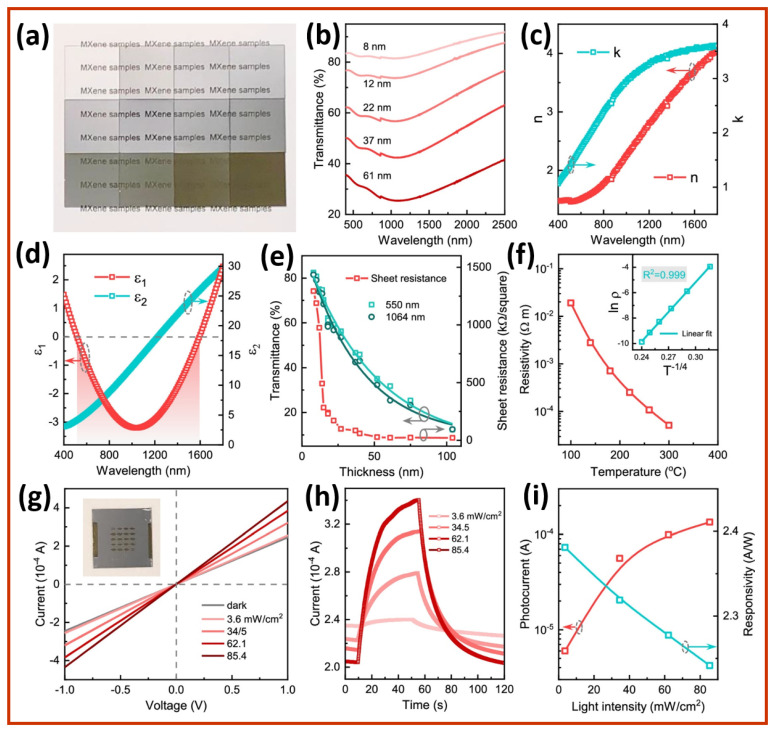
Optoelectronic characterization of Nb_2_CT_x_ MXene thin films for NIR photodetection. (**a**) Optical images of films with thicknesses from 8 to 61 nm. (**b**) UV–Vis–NIR spectra showing broad absorption with a peak at 1100 nm. (**c**) Ellipsometry of an 86 nm film reveals the refractive index (n) and extinction coefficient (k). (**d**) Real (ε_1_) and imaginary (ε_2_) parts of dielectric permittivity, with ε_1_ negative from 520–1590 nm, indicating plasmonic behavior. (**e**) Thickness-dependent sheet resistance and transmittance at 550 nm and 1064 nm. (**f**) Temperature-dependent resistivity showing non-metallic behavior. (**g**) I-V curves under varying 1064 nm laser intensities. (**h**) Temporal photoresponse with prolonged rise/decay times (>40 s/20 s) due to trap-dependent transport. (**i**) Photocurrent and responsivity (up to 2.3 A/W), limited by high dark current and low on/off ratio. Reproduced with permission from Ref. [90]. Copyright (2022) American Chemical Society.

The fundamental optoelectronic properties of Nb_2_CT_x_ MXene films are clearly illustrated in Figure 5a–i, as these properties are crucial for their functionality in next-generation self-powered photodetectors [90]. The optical absorption behavior of Nb_2_CT_x_ films appears through the combination of digital photos and UV–Vis–NIR transmittance spectra, as depicted in Figure 5a,b. Light transmittance deteriorates smoothly across the visible to near-infrared (NIR) spectrum as the film thickness increases from 8 to 61 nm. The photons interact strongly with the material, adhering to Beer–Lambert law principles. The material exhibits suitable characteristics for plasmonic near-infrared (NIR) photodetection, as evidenced by its 1100 nm NIR absorption peak, which signifies out-of-plane surface plasmon resonances (SPRs). Figure 5c presents the extinction coefficient (k) and refractive index (n) of the Nb_2_CT_x_ films that support the detection of broad-spectrum absorption. Figure 5d illustrates the complex dielectric permittivity (ε = ε_1_ + iε_2_), where ε_1_ turns negative from 520 to 1590 nm, providing clear evidence of the plasmonic behavior of the Nb_2_CT_x_ films. The negative real permittivity value indicates that surface plasmon oscillations contribute to efficient NIR hot-carrier generation. Research on the NIR plasmonic window in MXene-based devices remains significant, as previous studies on MXenes have primarily focused on visible-light operation. Figure 5e illustrates the trade-off between sheet resistance and optical transmittance concerning material thickness. Films thinner than 20 nm offer a high transparency but exhibit a sheet resistance more significant than 1 MΩ/sq. Meanwhile, thicker films, at 61 nm, provide a resistance of 21 kΩ/sq but lead to decreased optical transparency. This contrasting relationship highlights the essential trade-off between absorption and conductivity in the design of photodetectors. The temperature-dependent resistivity results shown in Figure 5f illustrate how Nb_2_CT_x_ MXene materials display non-metallic behavior, characterized by increasing resistivity rates during cooling periods. Analysis based on the 3D variable range hopping (VRH) model, illustrated in the inset, shows that charge carriers move locally due to the grain boundaries, chemical etching defects, and film porosity of the Nb_2_CT_x_. The Nb_2_CT_x_ device performance can be improved by reducing dark current and enhancing the on/off ratio by using a suitable blocking layer. Additionally, it is crucial to limit the carrier mobility behavior of the Nb_2_CT_x_. Planar Nb_2_CT_x_ devices generate photoresponses, as shown in Figure 5g,h, when stimulated with 1064 nm laser illumination. An increase in photoresponse from the device occurs in response to changes in laser intensity, reaching a value of ~10^4^ A at 62.1 mW/cm^2^. The Nb_2_CT_x_ device’s response time is slow because its rise and decay times are approximately 40 s, followed by a 20 s period. The Nb_2_CT_x_ devices exhibit low on/off ratio values of less than 2 due to their dark current being approximately 2.4 × 10^−4^ A. Furthermore, the Nb_2_CT_x_ photodetectors achieve a photoresponsivity of about 2.3 A/W. Plasmonic absorption generates sufficient hot carriers; however, a lack of effective charge-blocking technology results in excessive dark current, leading to poor signal-to-noise ratios in Nb_2_CT_x_ MXene photodetectors.

## 4. Gas Sensing Properties

Sensors made from MXene are significantly influenced by humidity, enhancing their conductivity and sensing capabilities. The surface groups of MXene (–OH, –F, –O) make it highly attractive to water, allowing it to adhere to the surface easily at a normal temperature and pressure. This adsorption enhances proton mobility and modifies the local charge, thus improving the MXene’s conductivity under moderate humidity. High humidity can cause the MXene coating to swell or detach, impeding electron movement and potentially resulting in signal drift. MXenes are more sensitive to moisture due to their varied surface chemistry and electronic structure, and this sensitivity can differ depending on the sensor design. Similarly, humidity affects temperature-dependent MXene sensors by altering the carrier concentration and charge at the interface. To ensure that MXene sensors are reliable for everyday use, it is essential to incorporate humidity compensation strategies, such as encapsulation or creating hydrophobic sensors.

Temperature fluctuations between 0 °C and 40 °C can significantly affect the conductivity and precision of MXene-based gas sensors. Within this range, increased thermal energy allows MXene charge carriers to move more freely, thereby enhancing electrical conductivity through electron hopping. Additionally, moderate heating enhances the adsorption and desorption of gases on the MXene surface, potentially improving the sensor’s sensitivity and response time. Nevertheless, these benefits face challenges due to the potential for signal drift caused by variations in conductivity and the interaction of the MXene sensor with the analyte at different temperatures. To accurately detect various gases in varying ambient conditions, the MXene sensor requires temperature calibration, real-time correction, and a reference sensor to manage fluctuations, ensuring smooth operation across a temperature range of 0 to 40 °C.

### 4.1. 4-Nitrophenol, Propofol, Serotonin, and NO_2_ Sensing

The Nb_2_CT_x_/Zn-Co-NC composite is an effective and selective electrochemical sensor for detecting 4-nitrophenol [96]. It is observed that incorporating Zn-Co-NC nanocages significantly enhances the electrochemical properties of Nb_2_CT_x_. The combination of Nb_2_CT_x_ and Zn-Co-NC boosts the conductivity, stability, and sensing activity of the sensor. The Nb_2_CT_x_/Zn-Co-NC sensor efficiently detects 4-nitrophenol in river and industrial wastewater over a detection range of 1 to 500 μM, with a low detection limit of 0.070 μM and a high sensitivity of 4.65 μA μM^−1^cm^−2^. Moreover, the non-covalent incorporation of Zn-Co-NC nanocages prevents the restacking of Nb_2_CT_x_, which increases the surface area and improves electron transport, showing excellent reproducibility, repeatability, and stability. Therefore, it is concluded that this work emphasizes the significant potential of thin Nb_2_CT_x_ MXenes combined with Zn-Co-NC composites for enhancing electrochemical detection technologies for toxic pollutants in future applications. Remarkably, a carbon cloth substrate-based electrochemical sensor combining SnO_2_ nanoparticles with Nb_2_CT_x_ MXene composite has been developed for propofol (PPF) detection [97]. With an impressive detection limit of 0.24 µM, the Nb_2_CT_x_ MXene/SnO_2_ sensor has a detection range extending from 1 to 300 µM. The synergistic properties of SnO_2_ and Nb_2_CT_x_ MXene facilitate improved electron transfer and increase the surface area, resulting in a high sensitivity and selectivity. The Nb_2_CT_x_ MXene/SnO_2_ sensor demonstrated an outstanding mechanical flexibility, selectivity against biological interferents, and reproducibility. Moreover, a prototype wearable patch was designed for point-of-care testing, achieving a detection limit of 0.436 µM within the concentration range of 1–100 µM. Therefore, the Nb_2_CT_x_ MXene/SnO_2_ sensor technology is a benchmark for future biosensing applications that will monitor anesthetic drugs in critical care through flexible, disposable frameworks. On the other hand, an electrochemical sensor for serotonin detection was investigated utilizing Nb_2_CT_x_ MXene nanocomposite structures combined with protonated carbon nitride (PCN) [98]. This study presents an enhanced method for serotonin detection that prioritizes sensitivity, selectivity, and cost-effectiveness, addressing the shortcomings of existing sensing techniques. By leveraging electrostatic interactions, the researchers created an Nb_2_CT_x_ MXene/PCN composite, enhancing its conductivity and reaction speed. The sensor functioned with a carbon cloth thread electrode, achieving a detection limit of 63.24 nM across a testing range of 1–100 μM while maintaining a high selectivity in the presence of interfering biomolecules. The Nb_2_CT_x_ MXene/PCN composite sensor demonstrated stable performance, and repeatability, suggesting its suitability as a disposable sensor. The Nb_2_CT_x_ MXene/PCN composite sensor performed effectively with spiked human serum, and its integration into a thread-based portable form enhances its practical usability. This research work marks a significant advancement in neurotransmitter detection, as Nb_2_CT_x_ MXene-PCN composites exhibit great promise for real-time biomedical diagnostics and wearable biosensing applications.

**Figure 6:**
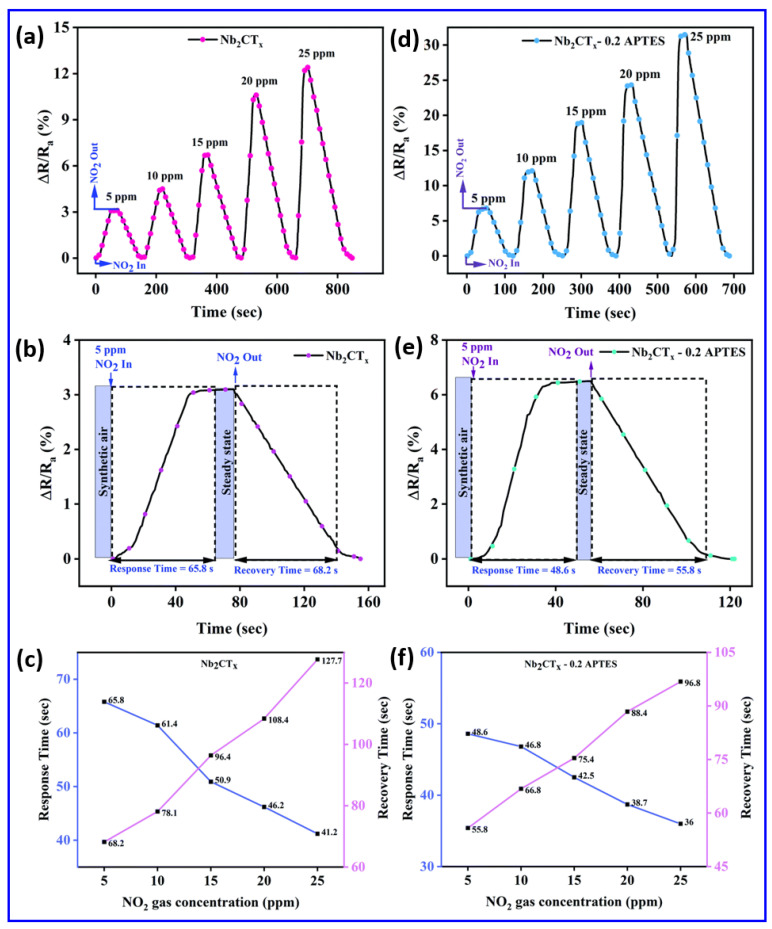
Comprehensive evaluation of the NO_2_ gas sensing performance of pristine Nb_2_CT_x_ MXene and APTES-functionalized Nb_2_CT_x_-0.2 APTES MXene at room temperature. (**a**,**d**) Dynamic response profiles (5–25 ppm NO_2_) reveal a significantly enhanced sensitivity for the functionalized sensor (31.52%) compared to the pristine Nb_2_CT_x_ MXene sensor (12.5%, attributed to the amine functional groups and the increased surface area). (**b**,**e**) Response–recovery kinetics at 5 ppm demonstrate faster adsorption and desorption in the functionalized Nb_2_CT_x_-0.2 APTES sensor, confirming improved interfacial charge transfer. (**c**,**f**) Response and recovery times decrease with higher NO_2_ levels, with consistently superior performance from the functionalized Nb_2_CT_x_-0.2 APTES sensor. Reproduced with permission from Ref. [99]. Copyright (2022) Royal Society of Chemistry.

One of the most critical aspects in the performance of room-temperature gas sensors is their sensitivity to ambient temperature fluctuations. Unlike high-temperature gas sensors that operate at 250–350 °C and incorporate microheaters to stabilize the sensing environment, room-temperature sensors lack such built-in thermal regulation mechanisms. This limitation introduces a significant challenge: even minor variations in room temperature can induce pronounced changes in the sensor’s electrical resistance, independent of gas exposure. This behavior arises from the intrinsic temperature dependence of conductivity, governed by factors such as carrier mobility, bandgap excitation, and surface adsorption/desorption dynamics. To address these challenges, researchers have turned to advanced material design to enhance the sensitivity and selectivity at room temperature while minimizing thermal drift. A compelling example is the APTES-functionalized Nb_2_CT_x_ MXene, which shows an enhanced NO_2_ response operating at room temperature, with superior sensitivity, as illustrated in Figure 6a–f [99]. Figure 6 indicates that APTES-functionalized Nb_2_CT_x_ MXene surpasses unaltered MXene in all aspects of sensing dynamics, together with response–recovery properties and concentration-dependent behavior. Figure 6a,d shows the transient response characteristics of the Nb_2_CT_x_ MXene and Nb_2_CT_x_-0.2 APTES sensors for various concentrations of NO_2_ at room temperature. It is observed that the Nb_2_CT_x_-0.2 APTES sensor displayed a significantly enhanced response of 31.52% compared to the 12.5% response of pristine Nb_2_CT_x_ MXene at 25 ppm NO_2_. The APTES modification results in a response increase exceeding twice the initial value, as it introduced amine (–NH_2_) functional groups into the Nb_2_CT_x_-0.2 APTES sensor. Enhanced interactions between electron-donating groups on the sensor surface and electron-withdrawing NO_2_ molecules improve the adsorption kinetics and charge transfer processes. The response recovery profiles in Figure 6b,e are further examined to emphasize the kinetic advantages of APTES functionalization in the Nb_2_CT_x_-0.2 APTES. It was observed that the Nb_2_CT_x_-0.2 APTES sensor had a faster response and relatively quicker recovery compared to the pristine sensor at 5 ppm NO_2_. Moreover, Figure 6c,f illustrates the response and recovery times of the Nb_2_CT_x_ MXene and Nb_2_CT_x_-0.2 APTES sensors at a 5 ppm NO_2_ concentration. Interestingly, the surface treatment of Nb_2_CT_x_ MXene with APTES (Nb_2_CT_x_-0.2 APTES) demonstrates its ability to optimize kinetic sensor behavior, improving the electron exchange pathway and increasing the rate of redox surface reactions for detecting NO_2_ gas.

### 4.2. Tea, Tyramine, and Humidity Sensing

Moreover, a novel SnS_2_/Nb_4_C_3_T_x_ MXene composite gas sensor was designed for the sensitive detection of the toxic volatile organic compound triethylamine (TEA) at room temperature [100]. This innovative research utilizes the impressive electrical conductivity and oxidation resistance of Nb_4_C_3_T_x_ MXene along with the outstanding gas adsorption properties of SnS_2_ nanosheets. The SnS_2_/Nb_4_C_3_T_x_ MXene sensor exhibited an exceptional TEA detection response of 1046.6% at a concentration of 50 ppm, with rapid response and recovery times of 11 s and 158 s, respectively. Remarkably, after 60 days of long-life exposure, the SnS_2_/Nb_4_C_3_T_x_ MXene sensor maintained a response exceeding 300%, indicating reliable operational performance. The in situ synthesis of SnS_2_ on Nb_4_C_3_T_x_ MXene provided a uniform distribution, resulting in an enhanced specific surface area and numerous active sites for gas interaction. The integrated structure of SnS_2_/Nb_4_C_3_T_x_ MXene overcame the challenges associated with stacking, promoting effective gas diffusion and efficient electron transport. Remarkably, the SnS_2_/Nb_4_C_3_T_x_ MXene sensor outperformed both metal oxide and MXene-based sensors under comparable operating conditions, which strongly supports the potential of Nb-based MXene composites as effective devices for TEA sensing applications at ambient temperatures, contributing to safer environmental monitoring and food quality assessments. Conversely, an Nb_2_CT_x_ MXene-assisted double S-tapered fiber-based LSPR sensor showed enhanced capabilities for tyramine detection [101]. This study harnesses the impressive attributes of Nb_2_CT_x_ MXene, including its extensive surface area, versatile surface chemistry, and superior electrical conductivity, to enhance localized surface plasmon resonance (LSPR) on double S-tapered fiber (DSF) platforms. The Nb_2_CT_x_ MXene sensor’s functionalization with gold nanoparticles and tyrosinase enables the selective measurement of tyramine, a biogenic amine related to food safety. New sensors featuring Nb_2_CT_x_ MXene have demonstrated a significantly improved performance compared to traditional graphene oxide devices, achieving twice the sensitivity (34 pm/µM) and a greater detection accuracy (0.35 µM) within a range of 0–300 µM. The DSF design enhances evanescent wave exposure, in combination with Nb_2_CT_x_ MXene’s plasmonic enhancement, leading to considerable improvements in light–matter interaction. The Nb_2_CT_x_ MXene sensor exhibits a high selectivity due to the specific enzymatic action of tyrosinase. An investigation has been conducted on enhancing MXene-based humidity sensors using few-layered Nb_2_CT_x_ MXene nanosheets [102]. A crucial discovery emerges from self-oxidative processing in aqueous solutions at steady temperatures throughout specified durations. This approach results in modifications to structure and chemistry, enabling a shift from electronic to ionic conduction mechanisms. The oxidation process generates new oxygen-containing functional groups that facilitate the adsorption of water molecules, thereby improving the humidity response. A humidity sensor utilizing optimally incubated Nb_2_CT_x_ MXene nanosheets attained an impressive sensitivity of −2.3 × 10^4^ at a 53% relative humidity after 27 days of incubation at room temperature. This study offers valuable insights into the oxidation processes of Nb_2_CT_x_ MXene and related sensing techniques, facilitating the creation of highly sensitive, selective, and flexible humidity sensors that can be practically applied in real-world applications. A novel sensor design was presented, incorporating layered BTO-doped P(VDF-TrFE) active piezoelectric materials within PDMS-containing conductive Nb_2_CT_x_ MXene [103]. This groundbreaking design tackles the persistent challenges of filler distribution and accurate dipole orientation. The sensor enhances piezoelectric output by the careful placing of a conductive interlayer, which leads to a dipole alignment that is perpendicular to the film surface, influenced by interfacial polarization. With a 6.2 μm thick interlayer, the sensor demonstrates an impressive sensitivity of 16.05 V/N and a quick response time of 626 μs. Therefore, it is concluded that this research work sets an industry standard for developing self-powered sensors, while outlining a viable pathway for both wearable electronics and IoT applications.

**Figure 7:**
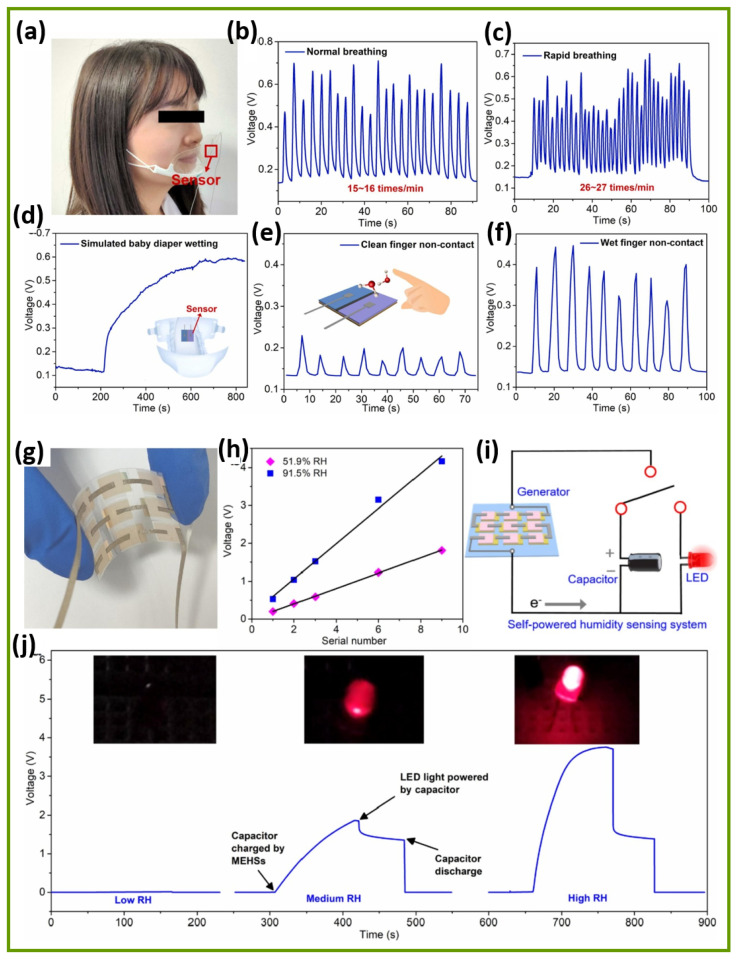
This demonstrates the versatile applications of the Nb_2_CT_x_/HA-based primary battery humidity sensor (PBHS). (**a**–**f**) The integration of the PBHS into a commercial face mask enables the real-time monitoring of respiration. Steady voltage oscillations are observed at a rate of 15–16 breaths per minute during normal breathing. At a faster pace of 26–27 breaths per minute, the PBHS device successfully demonstrates proper voltage response patterns. During diaper wetting, the PBHS achieves a stable voltage of 0.6 V, showcasing its effectiveness for innovative hygiene monitoring applications. A dry finger engages in non-contact detection and results in moderate voltage fluctuations near the PBHS until the finger is removed. The detection system generates higher voltage outputs from wet fingers, suggesting potential applications in hygienic touchless switching. Reproduced with permission from Ref. [104]. Copyright (2023) Elsevier. Furthermore, a self-powered humidity sensing system is realized through the Nb_2_CT_x_ MXene/SA composite-based MEHS array. (**g**) The 3 × 3 MEHS array is depicted in the image; (**h**) demonstrates that voltage output increases as additional devices are connected in series, (**i**) includes a circuit diagram of the MEHS array combined with a capacitor and an LED, and (**j**) presents visual humidity detection across low, medium, and high RH levels, with varying LED brightness levels indicating humidity without external power. Reproduced with permission from Ref. [105]. Copyright (2022) Elsevier.

The integration of hyaluronic acid (HA)-induced crumpling Nb_2_CT_x_ MXene nanosheets into a primary battery-based humidity sensor (PBHS) demonstrates significant multifunctional applications, as illustrated in Figure 7a–f [104]. It suggests the Nb_2_CT_x_ MXene sensor’s versatility, response sensitivity, practical feasibility, and broader implications for innovative sensing technologies. The Nb_2_CT_x_ MXene/HA-based PBHS device shows extraordinary sensitivity, together with quick response capabilities, in the measurement of human breathing patterns, as shown in Figure 7a–c. The Nb_2_CT_x_ MXene/HA-based PBHS sensor operates effectively as an integrated part of commercial face masks to monitor changes in exhaled air moisture. The Nb_2_CT_x_ MXene/HA-based PBHS sensor device generates signals with a changing output between 0.4 V and 0.7 V when operated under typical breathing patterns of 15 to 16 breaths per minute. The Nb_2_CT_x_ MXene/HA-based PBHS sensor responds with additional high-frequency voltage peaks when air moisture monitoring occurs at fast respiratory rates between 26 and 27 breaths per minute. In addition, the Nb_2_CT_x_ MXene/HA-based PBHS sensor demonstrates advantages for real-time health applications, especially in care facilities when monitoring patients with asthma and COPD, along with the exercise observation of athletes. The self-powered configuration of the Nb_2_CT_x_ MXene eliminates the necessity of an external power supply, which makes it highly suitable for wearable health device applications. Figure 7d demonstrates the Nb_2_CT_x_ MXene/HA-based PBHS’s capability to sense progressive humidity growth, which models conditions of diaper wetness. Remarkably, moisture increases the Nb_2_CT_x_ MXene/HA-based PBHS’s voltage continuously until it approaches a stable state at 0.6 V. It can provide an effective tool for continuous baby care and adult urinary care for elderly persons with incontinence issues. Interestingly, the early identification of moisture allows for the interruption of wet conditions before they develop, thereby promoting skin health and hygienic conditions without the risk of irritation or infection. Traditional diaper sensors need battery replacements or external recorders for their operation. However, the energy-autonomous system design reduces the complexity of usage and maintenance needs. The Nb_2_CT_x_ MXene/HA-based PBHS shows changes operating at different moisture levels, as shown in Figure 7e,f. The Nb_2_CT_x_ MXene/HA-based PBHS detects moderate voltage spikes when a clean (dry) finger draws near due to its baseline sensitivity to humidity changes, as depicted in Figure 7e. The Nb_2_CT_x_ MXene/HA-based PBHS generates stronger voltages after exposure to a wet finger, as this method provides a better surface conductivity because of added moisture, as illustrated in Figure 7f. The Nb_2_CT_x_ MXene/HA-based PBHS demonstrates potential usefulness for public health situations that require touchless controls, since these applications must reduce the spread of bacteria and viruses during emergencies. Therefore, detecting moisture levels creates opportunities to develop technologies that can be useful for gesture recognition and authentication systems in the near future. The Nb_2_CT_x_ MXene/HA-based PBHS presents a power-efficient, environmentally friendly operation as an alternative. The process-based evaluation of Nb_2_CT_x_ MXene/HA-based PBHS demonstrates its ability to function as a flexible, humidity-sensitive, and innovative system. The Nb_2_CT_x_ MXene/HA-based PBHS device combines self-charging properties with a quick 15.1 s response time and a fast recovery time of 3.4 s while producing lasting power generation up to 12 h, making it ready for real-world use. Moreover, integrating energy harvesting technology directly within sensor architecture represents a vital research priority for developing fully autonomous humidity detection systems. Nb_2_CT_x_ MXene/sodium alginate (SA) composite-based moisture–electric humidity sensors (MEHS) demonstrate a strong potential, offering quick response times, a high sensitivity, and operational power generation capabilities for self-powered humidity detection, as elucidated in Figure 7g–j [105]. Figure 7g–j show a complete demonstration of Nb_2_CT_x_ MXene/SA composite-based MEHS for real-life situations [105]. The figure demonstrates how the Nb_2_CT_x_ MXene/SA composite-based MEHS array joins with a standalone power supply to create an autonomous humidity sensor that overcomes the ongoing problem of external power requirements in wearable devices and sensors. A 3 × 3 array of Nb_2_CT_x_ MXene/SA composite-based MEHS was ingeniously designed to enhance its voltage response, as demonstrated in Figure 7g,h. It is observed that the total output voltage increased to nearly 4.17 V for the 3 × 3 array series connection through the systematic linking of individual Nb_2_CT_x_ MXene/SA composite-based MEHS units under high relative humidity conditions (91.5% RH), as revealed in Figure 7h. The flexible system uses a modular approach to allow scalability, through which the output adjusts to meet application requirements. The authors made the system more practical by combining the Nb_2_CT_x_ MXene/SA composite-based MEHS array, which produced the self-powered sensing circuit, as shown in Figure 7i. This integrated system employs the capacitor to collect electrical power produced by the Nb_2_CT_x_ MXene/SA composite-based MEHS array, activating the LED, which creates an autonomous humidity detection setup since it requires no additional external power supply. Figure 7j successfully depicts the integrated Nb_2_CT_x_ MXene/SA composite-based MEHS’s reaction to changing humidity conditions through its impact on LED brightness states. The Nb_2_CT_x_ MXene/SA composite-based MEHS generates little voltage output in low humidity conditions, which results in a weak LED illumination. On the other hand, the LED’s brightness becomes increasingly intense as humidity levels rise at ~59% RH and ~97% RH, showing real-time visual evidence of humidity variation. This demonstration holds great value by confirming that the Nb_2_CT_x_ MXene/SA composite-based MEHS can function successfully as both a powerful humidity sensor and renewable power generator. Therefore, these technological innovations present substantial promise for different implementation areas, since they can be used in wearable health monitoring devices along with environmental sensors, smart packaging systems, and safety alert devices during situations where battery maintenance is difficult.

### 4.3. Pressure, H_2_, Methanol, and NH_3_ Sensor

A multifunctional PAA/PEDOT:PSS/Nb_2_CT_x_ MXene hydrogel explored a new standard for flexible pressure sensors due to its remarkable mechanical, conductive, and self-healing properties [106]. The hydrogel combines Nb_2_CT_x_ MXene nanosheets with PEDOT:PSS in a dual-network framework, enabling a stretchability of 462.2%, toughness of 120.71 kJ/m^3^, and conductivity of 165.54 S/m. The integration of these components allows the PAA/PEDOT:PSS/Nb_2_CT_x_ MXene sensor to achieve extreme sensitivity with a 9.81 GF rating, but it also possesses a minimal pressure threshold detection at 99.87 Pa, along with a quick reaction speed and recovery time of 200 ms across 2000 cycles. After glycerin treatment, the hydrogel maintains its functionality at sub-zero temperatures because of its effective anti-freezing characteristics. The PAA/PEDOT:PSS/Nb_2_CT_x_ MXene hydrogel demonstrates excellent surface attachment properties with human skin, while preserving its self-healing ability at a more than 80% capability without significant functional losses. It also boasts several features that address conventional design problems of piezoresistive sensors by offering better ductility, superior electrical properties, and lower sensitivity to external factors, thus establishing a robust framework for wearable devices. A novel hydrothermal method generates hydrogen fluoride in situ and produces multilayer Nb_2_CT_x_ MXene with effective hydrogen gas detection at room temperature [107]. The novel synthesis method employed LiF in combination with NH_4_F and HCl to enhance laboratory safety and achieve more accurate process conditions, moving away from traditional HF etching techniques. The interlayer spacing in LiF-etched Nb_2_CT_x_ MXene material increased because Li^+^ possesses a larger hydration radius, which allows for improved gas accessibility without the need for further exfoliation processes. The detection sensor made from LiF-Nb_2_CT_x_ showed sharp response–recovery properties when detecting various gases at very low concentrations down to 0.5 ppm, including hydrogen and ammonia. This study highlights the effective application of Nb_2_CT_x_ MXene/Nb_2_O_5_ as a temperature-efficient gas sensor. It shows variations in interlayer gaps and surface types, which can influence the gas sensing performance of the sensor, furthermore developing an advanced methanol gas sensor through the nanocomposite synthesis of Ti_3_C_2_T_x_-BaSnO_3_-Nb_2_CT_x_ (TC-BSO-Nb-1) using the hydrothermal method [108]. Methanol sensing performance improvement in the TC-BSO-Nb-1 nanocomposite occurred due to the combined effects of individual materials (layered MXenes with BaSnO_3_ nanorods). The TC-BSO-Nb-1 sensor exhibited high resistance to 50 ppm methanol detection at 110 °C, achieving a remarkable 98% response rate. It demonstrated outstanding selectivity to volatile organic compounds and long-term stability over 28 days while maintaining low detection limits at 0.92 ppb. The exceptional performance of the TC-BSO-Nb-1 sensor is illustrated by its 74.51 m^2^/g specific surface area, abundant oxygen vacancies, optimized electronic band structure, and heterojunction and Schottky interfaces. Among all the examined sensors, the TC-BSO-Nb-1 sensor offered both a lower operation temperature and improved detection thresholds, which make it suitable for real methanol monitoring applications.

**Figure 8:**
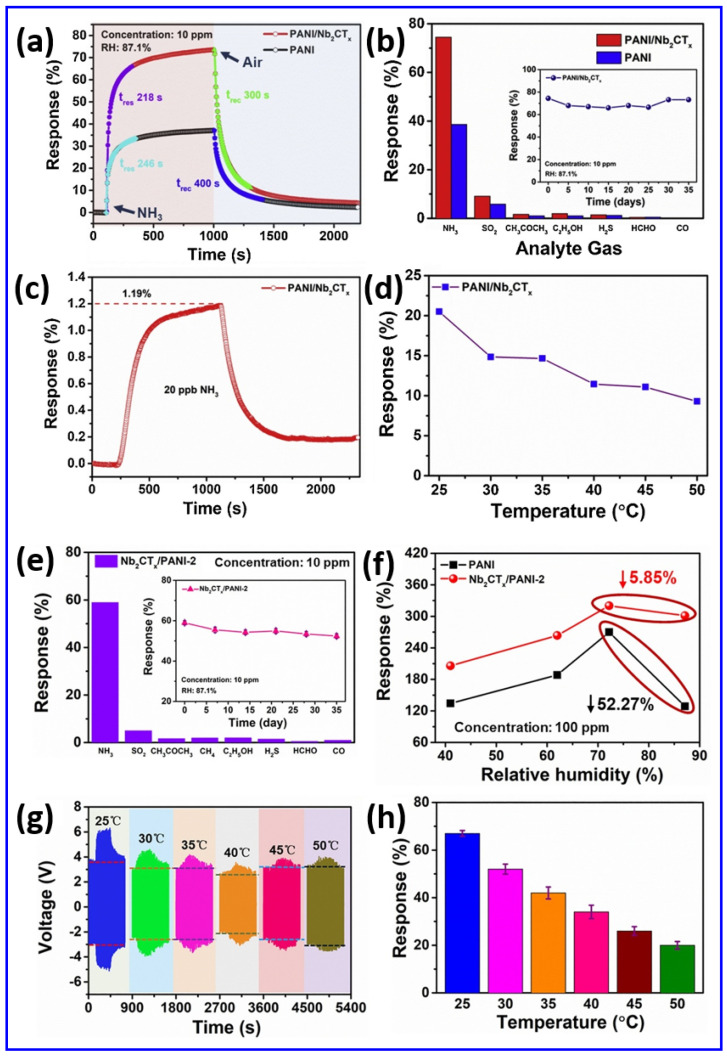
Comprehensive gas sensing performance of PANI/Nb_2_CT_x_ MXene nanocomposite sensor under high humidity conditions (87.1% RH). (**a**) Response and recovery curves of PANI and PANI/Nb_2_CT_x_ MXene sensors to 10 ppm NH_3_, with Nb_2_CT_x_ MXene nanocomposite aiding in the regulation of the PANI morphology and hence improving its response and recovery kinetics. (**b**) The PANI and PANI/Nb_2_CT_x_ MXene composite sensors showed high selectivity towards detecting NH_3_ as they differentiated it from common interfering gases (inset demonstrates PANI/Nb_2_CT_x_ MXene sensor’s sensing of 10 ppm NH_3_). (**c**) The PANI/Nb_2_CT_x_ MXene composite sensor demonstrates an exceptional capability to detect NH_3_ at concentrations as low as 20 ppb, which makes the sensor suitable for monitoring trace-level substances in breath and environmental conditions. (**d**) Varying the PANI/Nb_2_CT_x_ MXene composite sensor operating temperature from 25 °C to 50 °C led to a reduced sensitivity to 2 ppm NH_3_ because the gas was evaporated at higher speeds (due to more energetic desorption dynamics). Reproduced with permission from Ref. [109]. Copyright (2021) Elsevier. (**e**) The selectivity profile of the Nb_2_CT_x_ MXene/PANI-2 sensor towards various gases at 87.1% relative humidity (RH) shows a highly selective response to NH_3_ (10 ppm), with a response at least 11.79 times greater than that of the other tested gases. The inset illustrates the long-term stability of the sensor, which retains 89% of its initial response over 35 days. (**f**) The influence of varying humidity levels (40–90% RH) on the NH_3_ sensing response (100 ppm) of pure PANI and Nb_2_CT_x_ MXene/PANI-2 sensors highlights the superior humidity tolerance of Nb_2_CT_x_ MXene/PANI-2, attributed to intermolecular hydrogen bonding that limits water adsorption. (**g**) The dynamic response and recovery behavior of the Nb_2_CT_x_ MXene/PANI-2 sensor towards 10 ppm NH_3_ under different operating temperatures (25–50 °C) at 87.1% RH indicates a temperature-dependent decrease in response due to enhanced desorption at higher temperatures. (**h**) The quantitative gas sensing response of the Nb_2_CT_x_ MXene/PANI-2 sensor towards 10 ppm NH_3_ across a temperature range of 25–50 °C under 87.1% RH demonstrates a reliable performance even at elevated temperatures, making it suitable for practical applications. Reproduced with permission from Ref. [110]. Copyright (2021) Elsevier.

Figure 8a–d assesses all the essential features, including dynamic performance, selectivity, stability, ultra-low concentration detection, and temperature dependence of the PANI/Nb_2_CT_x_ MXene for NH_3_ sensor for practical applications in exhaled human breath under high-humidity conditions [109]. Figure 8a compares the dynamic response of PANI and PANI/Nb_2_CT_x_ MXene sensors when measuring 10 ppm NH_3_ at 87.1% RH. The PANI/Nb_2_CT_x_ MXene sensor shows response and recovery times surpassing PANI alone. The rapid response and recovery kinetics for NH_3_ adsorption/desorption under humid conditions are due to the controlled morphology of PANI nanofibers influenced by the synergistic effects of Nb_2_CT_x_ MXene nanosheets. The PANI/Nb_2_CT_x_ MXene sensor demonstrates exceptional selectivity for detecting NH_3_, as shown in Figure 8b, because it exhibits a better response to NH_3_ under various interfering gases such as CH_4_, C_2_H_5_OH, C_3_H_6_O, H_2_, and CO. This unique selectivity capability of the PANI/Nb_2_CT_x_ MXene sensor is crucial for the sensor, particularly in real-world environments containing various volatile compounds found in exhaled breath and industrial facilities. The strong chemical bond between the surface functional groups (–OH, –O) of Nb_2_CT_x_ MXene sensor nanosheets and NH_3_ molecules, coupled with the p-n heterostructuring of the PANI/Nb_2_CT_x_ MXene sensor nanocomposite, enhances its selectivity for NH_3_ detection while minimizing cross-interactions with other vapor molecules. The inset of Figure 8b demonstrates that the PANI/Nb_2_CT_x_ MXene sensor exhibits excellent stability during operation, showing a minimal variation of less than 11% over 35 days. Figure 8c illustrates that the PANI/Nb_2_CT_x_ MXene sensor exhibits a high sensitivity to NH_3_ detection at very low concentrations of 20 ppb while operating at 87.1% RH. The PANI/Nb_2_CT_x_ MXene sensor’s functionality at 20 ppb NH_3_ concentrations makes early medical diagnosis possible through breath analysis, aiding in chronic kidney disease screening and environmental monitoring. The hierarchical porous structure, combined with a high surface area in the composite, provides many active sites for interaction with gases, thus enabling an enhanced sensitivity at low concentrations. Figure 8d evaluates the performance of the PANI/Nb_2_CT_x_ MXene sensor under various operational temperatures, ranging from 25 to 50 °C. It is observed that the PANI/Nb_2_CT_x_ MXene sensor’s response mechanism decreases with increasing operating temperatures. The PANI/Nb_2_CT_x_ MXene sensor’s response diminishes because higher temperatures accelerate desorption processes, which results in limited overall adsorption, consequently leading to a reduced sensor response. The response–performance relationship based on temperature underscores the necessity of temperature monitoring in practical breath analysis, as the temperature of exhaled breath varies with environmental factors and states of human health. Additionally, the Nb_2_CT_x_ MXene/PANI-2 sensor demonstrates ammonia gas sensing capabilities, enhancing industrial and workplace safety, as illustrated in Figure 8e–h [110]. Figure 8e highlights the remarkable selectivity of the Nb_2_CT_x_ MXene/PANI-2 sensor towards ammonia (NH_3_) at an 87.1% relative humidity (RH). When exposed to 10 ppm NH_3_, the Nb_2_CT_x_ MXene/PANI-2 sensor’s response was significantly higher than its response to other interfering gases, including SO_2_, CH_3_COCH_3_, CH_4_, CH_3_OH, H_2_S, and HCHO. Notably, the response to NH_3_ was at least 11.79 times greater than that to any other tested gas, underscoring its high specificity for ammonia detection. This exceptional selectivity can be attributed to the synergistic sensing properties of PANI, which possesses an inherent selectivity towards NH_3_, and the MXene (Nb_2_CT_x_) component with abundant surface terminations that strongly interact with NH_3_ molecules. Furthermore, the inset of Figure 8e demonstrates the Nb_2_CT_x_ MXene/PANI-2 sensor’s long-term stability, maintaining 89% of its initial response over 35 days under humid conditions (87.1% RH). Figure 8f illustrates the effect of varying humidity levels on the NH_3_ sensing response of pure PANI and the Nb_2_CT_x_ MXene/PANI-2 sensor at 100 ppm. Within the typical humidity range (41.0–87.1% RH), both sensors exhibited an initial increase in response as humidity increased, likely due to the hygroscopic nature of NH_3_ promoting its adsorption. However, at a very high humidity (87.1% RH), the response began to decline, indicating that water molecules occupy active adsorption sites, thus hindering NH_3_ adsorption. Notably, the Nb_2_CT_x_ MXene/PANI-2 sensor showed a significantly lower sensitivity loss at high humidity than pure PANI. The response attenuation of the Nb_2_CT_x_ MXene/PANI-2 sensor was 8.94 times lower than that of the PANI sensor, which can be attributed to the formation of intermolecular hydrogen bonds between PANI and Nb_2_CT_x_ MXene. These bonds effectively occupy water adsorption sites, mitigating the adverse effects of humidity and enhancing the sensor’s robustness in humid environments. Figure 8g examines the dynamic response and recovery behavior of the Nb_2_CT_x_ MXene/PANI-2 sensor at various temperatures (25–50 °C) under 87.1% RH while detecting 10 ppm NH_3_. The results reveal a clear trend; as the temperature increases, the Nb_2_CT_x_ MXene/PANI-2 sensor’s voltage response decreases. This inverse relationship can be attributed to the desorption dynamics of NH_3_ at higher temperatures, leading to a reduction in the number of adsorbed molecules on the Nb_2_CT_x_ MXene/PANI-2 sensor’s surface. Despite this decline, the Nb_2_CT_x_ MXene/PANI-2 sensor still exhibits an appreciable responsiveness even at 50 °C, maintaining a significant response of around 20%. Such resilience indicates that the Nb_2_CT_x_ MXene/PANI-2 sensor can operate effectively across practical temperatures, making it suitable for breath monitoring (typically around 34–37 °C) and agricultural ammonia detection. Finally, Figure 8h quantifies the NH_3_ sensing response of the Nb_2_CT_x_ MXene/PANI-2 sensor across various temperatures. Figure 8h shows that the response decreases as the temperature increases from 25 °C to 50 °C. Notably, even at the highest tested limit (50 °C), the Nb_2_CT_x_ MXene/PANI-2 sensor maintains significant functionality. This result underscores the importance of considering temperature effects in real-world applications to ensure accuracy. Therefore, these findings affirm the Nb_2_CT_x_ MXene/PANI-2 sensor as a versatile and reliable platform for NH_3_ detection.

### 4.4. Hemoglobin, Sulfamethoxazole, Nonenzymatic Glucose, and Acetone Sensor

The use of Nb_2_CT_x_ MXene in microfiber-based biosensors has considerably enhanced hemoglobin detection, due to its high surface area, hydrophilicity, and outstanding photothermal properties [111]. The light-controlled optical method enhances light–matter interaction strength and signal enhancement, achieving an impressive sensitivity with ultra-low detection. The identified sensitivity rate exceeds normal physiological limits, indicating strong diagnostic potential. The Nb_2_CT_x_ MXene in microfiber-based biosensors can attribute its versatility in diverse applications to its resistance to electromagnetic interference and cost-effectiveness. This makes it suitable for clinical diagnostics, food safety, and environmental monitoring. Currently, there exists an advanced electrochemical detection method based on MIP-SiO_2_@Nb_2_CT_x_, which brings together few-layer Nb_2_CT_x_ structures with molecularly imprinted polymers to develop the selective electrochemical detection of sulfamethoxazole (SMX) [112]. The MIP-SiO_2_@Nb_2_CT_x_ sensor obtains improved conductivity together with a large surface area through the application of alkenyl ferrocene as a cross-linker. The signal ratio performance of the MIP-SiO_2_@Nb_2_CT_x_ sensor exceeds that of traditional MIP sensors and non-imprinted devices. Therefore, a successful wastewater analysis demonstrates that the MIP-SiO_2_@Nb_2_CT_x_ sensor has valuable applications for the environmental monitoring of pharmaceutical pollutants. Fascinatingly, the Nb_2_CT_x_—selenium nanoparticle composite shows excellent prospects as a nonenzymatic glucose sensor, while answering the worldwide requirement for better diabetes monitoring technologies [113]. The Nb_2_CT_x_–selenium nanoparticle composite demonstrates potential for developing reliable enzyme-free glucose monitoring devices, which can detect glucose concentrations from 2 to 30 mM, with a detection limit reaching 1.1 mM. Moreover, Nb_2_CT_x_ MXene has emerged as a promising candidate for relative humidity (RH) sensing due to its excellent photoelectronic and structural characteristics. Integrating Nb_2_CT_x_ MXene nanosheets with optical microfibers via an optical deposition method enables a fiber-optic RH sensor with dual sensitivity regions. Experimental results demonstrated a blue shift in transmission spectra from 18.5% to 72.4% RH due to refractive index modulation, and a red shift from 72.4% to 95.4% RH linked to structural changes. While Nb_2_CT_x_-based gas sensors boast remarkable gas detection capabilities, their responsiveness to oxidation and moisture greatly restricts their practical applications. Future studies should aim at creating a more stable environment for the reliable long-term operation of Nb_2_CT_x_-based gas sensors. Potential approaches include enhancing the surface hydrophobicity through polymers, utilizing thin oxide coatings via ALD for the material’s protection, or combining it with graphene or other 2D materials. Additionally, introducing dopants or alloying Nb_2_CT_x_ with various transition metals may enhance its resistance to redox reactions. By implementing these strategies, alongside safe device manufacturing and secure packaging, we can create robust Nb_2_CT_x_ MXene-based gas sensors.

**Figure 9:**
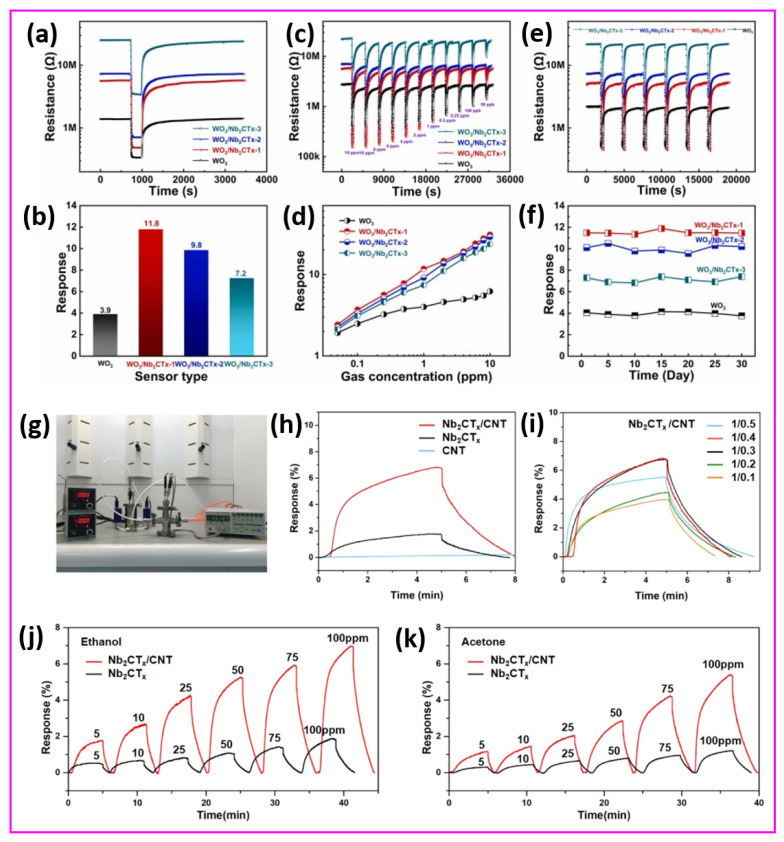
The acetone gas sensing responses of sensors fabricated using WO_3_, WO_3_/Nb_2_CT_x_-1, WO_3_/Nb_2_CT_x_-2, and WO_3_/Nb_2_CT_x_-3. (**a**) The resistance of sensors showed a dynamic change when exposed to 1 ppm acetone vapor at 200 °C, (**b**) selective response values of different sensors at 1 ppm acetone, (**c**) dynamic resistance measurement was monitored at different acetone concentration levels from 50 ppb to 10 ppm, (**d**) the response values vs. acetone concentration, (**e**) cyclic stability assessment under repeated exposure to 1 ppm acetone, and (**f**) long-term stability evaluation of the sensors over 30 days at 200 °C. Reproduced with permission from Ref. [114]. Copyright (2021) Elsevier. (**g**) A photograph shows the VOC sensing setup featuring mass flow controllers (MFCs), a gas chamber, and a data acquisition (DAQ) system. (**h**) The hybrid material Nb_2_CT_x_/CNT achieved a better sensitivity than Nb_2_CT_x_ and CNT-based sensors after exposure to 100 ppm ethanol vapors. (**i**) The Nb_2_CT_x_/CNT sensor exhibited its best performance for detecting 100 ppm ethanol when having a 1:0.4 ratio between Nb_2_CT_x_ and CNT. (**j**) Pristine Nb_2_CT_x_ and Nb_2_CT_x_/CNT hybrids demonstrated a better sensitivity and linear response over different ethanol concentration ranges from 5 to 100 ppm during evaluation. (**k**) The acetone detection performance of Nb_2_CT_x_ and Nb_2_CT_x_/CNT hybrid sensors over different acetone concentration ranges from 5 to 100 ppm. Reproduced with permission from Ref. [115]. Copyright (2024) American Chemical Society.

Figure 9a–f illustrate that WO_3_/Nb_2_CT_x_ heterojunction sensors produce improved acetone sensing results, attributed to their capability to enhance charge movement and expand active zones, while also showing greater durability [114]. Combining Nb_2_CT_x_ MXene with the WO_3_ structure enhances sensors’ sensitivity and rapid response time while maintaining excellent long-term operational stability. The WO_3_/Nb_2_CT_x_ heterojunction is valuable for future advancements in acetone gas sensors, particularly in systems that demand high environmental adaptability, sensitivity, and selectivity. Figure 9a shows that WO_3_ nanoparticles significantly improve acetone sensing performance when combined with Nb_2_CT_x_ MXene. The WO_3_/Nb_2_CT_x_ heterojunction sensor demonstrates a higher response during acetone detection, because its dynamic resistance transitions exceed those of pure WO_3_ sensors. The heterojunction interface of the WO_3_/Nb_2_CT_x_ creates a synergistic effect that facilitates improved electron transfer processes and quicker kinetics for gas adsorption and desorption. Monitoring the response and recovery of the WO_3_/Nb_2_CT_x_ heterojunction sensor, as shown in Figure 9b, is essential for evaluating its practical usefulness. The sensor utilizing WO_3_/Nb_2_CT_x_-1 reacts to acetone exposure in just 8 s, outperforming the pure WO_3_ sensor, which requires 11 s for the same reaction. The introduction of Nb_2_CT_x_ MXene enhances fast electron transfer processes, improving the interaction between acetone and oxygen species adsorbed on the surface. The recovery process for WO_3_ is longer than that of WO_3_/Nb_2_CT_x_-1, with recovery times of 707 s and 934 s, respectively, indicating that the desorption of acetone molecules takes additional time. Surface modification optimizes these processes, as acetone demonstrates strong binding properties with the WO_3_ and Nb_2_CT_x_ MXene. The sensing properties of the WO_3_/Nb_2_CT_x_ heterojunction sensor are illustrated in Figure 9c, which highlights their response patterns that change with varying acetone concentration levels. The WO_3_/Nb_2_CT_x_-1 sensor can detect low acetone concentrations, with a calculated detection limit of 0.8 ppb, making it suitable for healthcare applications like diabetes breath analysis tests. The sensors based on WO_3_/Nb_2_CT_x_ combinations perform better than WO_3_ sensors for all acetone concentrations, as shown in Figure 9d. The WO_3_/Nb_2_CT_x_-1 sensor shows fast reaction rates toward trace acetone detection, which makes it suitable for medical breathing analysis. Furthermore, the experimental results show that the material engineering in sensor development helps develop WO_3_/Nb_2_CT_x_-1 as a top choice for detecting acetone at low levels. The WO_3_/Nb_2_CT_x_ heterojunction sensor provides reliable readings throughout its cyclic stability tests, illustrated in Figure 9e. The WO_3_/Nb_2_CT_x_-1 sensor exhibits strong reliability throughout multiple operation cycles, demonstrating high structural and chemical durability. Interestingly, healthcare monitoring equipment requires sensors to demonstrate repeatable performance, which makes these sensors practical for continuous use. The long-term stability of the WO_3_/Nb_2_CT_x_-1 heterojunction sensor’s evaluation occurs during the 30 day testing period in Figure 9f. The combination of WO_3_ with Nb_2_CT_x_ into a heterostructure leads to both an improved sensor ability and environmental operational stability, as shown in Figure 9a–f. Acetone detection has advanced toward early diagnosis through portable breathing analysis systems focused on managing diabetes and other metabolic disorders. The VOC sensing capabilities of Nb_2_CT_x_/CNT hybrid material are evaluated in Figure 9g–k, which provides vital information about performance enhancement [115]. Figure 9g provides the precise delivery of gases with resistance measurements, thus generating reliable gas sensing properties. A robust configuration is essential in determining the performance levels of all sensors, including pristine CNT devices together with Nb_2_CT_x_ materials and hybrid Nb_2_CT_x_/CNT hybrid sensors. The Nb_2_CT_x_/CNT hybrid sensor demonstrates superior performance compared to the other sensors when exposed to ethanol, as revealed in Figure 9h. The individual CNTs function as passive conductive fillers, because they demonstrate minimal responsiveness, although Nb_2_CT_x_ shows moderate activity. The response of the Nb_2_CT_x_/CNT hybrid sensor surpasses the response of pristine Nb_2_CT_x_ by approximately 6.8%. The improved behavior demonstrates that adding CNTs efficiently prevents the restacking of the Nb_2_CT_x_ MXene, which makes more active surface sites and reaction areas accessible to VOC gas molecules. The combination of the high conductivity of CNTs and functional surface groups of the Nb_2_CT_x_ MXene generates an exceptional VOC gas sensing performance. Figure 9i tests the VOC gas sensing properties using the various ratios of Nb_2_CT_x_ MXene/CNT. It shows a maximum VOC response of a 1/0.4 weight ratio of Nb_2_CT_x_ MXene/CNT, after which it reduces with increasing CNT concentrations in Nb_2_CT_x_ MXene/CNT. The Nb_2_CT_x_ MXene/CNT hybrid sensor shows an excellent response for all 5 to 100 ppm ethanol concentrations compared with bare Nb_2_CT_x_ MXene, as depicted in Figure 9j. Figure 9k illustrates the acetone sensing response for 5 ppm to 100 ppm concentrations. The Nb_2_CT_x_ MXene/CNT hybrid sensor remains more sensitive throughout all acetone concentration tests when compared to the pure Nb_2_CT_x_ MXene sensor. Interestingly, the Nb_2_CT_x_ MXene/CNT hybrid sensor detects acetone more effectively by 5.6 times than the pure Nb_2_CT_x_ MXene sensor. It is concluded that Figure 9g–k shows that CNTs’ incorporation with Nb_2_CT_x_ MXene enhances its VOC sensing characteristics. Adding CNTs to Nb_2_CT_x_ MXene yields a superior gas detection performance because the modified Nb_2_CT_x_ MXene/CNT hybrid sensor has more active surface areas and better electrical conduction.

Within the growing family of MXenes, Nb_2_CT_x_ is recognized as a potential candidate for sensing applications, yet it remains less studied than the extensively researched Ti_3_C_2_T_x_ and V_2_CT_x_. Nb_2_CT_x_ exhibits notable sensitivity benefits due to the different oxidation states of niobium (Nb^3+^, Nb^4+^, Nb^5+^), enabling robust charge transfer interactions with target gases, thereby enhancing its gas sensing performance. Furthermore, its hydrophilic characteristics make it susceptible to moisture absorption, which may increase its recovery time and lead to signal instability in humid environments. Ti_3_C_2_T_x_ enjoys a well-established surface chemistry and tunable terminations such as –OH, –F, and =O; however, Nb_2_CT_x_ encounters challenges regarding the control of functionalization, potentially limiting its sensitivity optimization across various sensing platforms. Conversely, Ti_3_C_2_T_x_ sensors usually demonstrate quicker and more reliable response and recovery cycles due to enhanced processing and intercalation. In contrast, V_2_CT_x_ exhibits a rapid response but has a slower recovery due to its prolonged adsorption/desorption process. Stability is crucial for effective sensor technology, and here, Nb_2_CT_x_ offers benefits over V_2_CT_x_, especially in terms of oxidation resistance. The Nb–C bond ensures structural strength and improved ambient stability, making Nb_2_CT_x_ more suitable for long-term use. Nonetheless, its prolonged exposure can result in the creation of surface oxide, such as Nb_2_O_5_, which potentially reduces the electronic conductivity and sensor’s functionality. Furthermore, there is still limited long-term research on the cycling and reusability of Nb_2_CT_x_ sensors, which leaves a gap in our understanding of their durability in the real world. However, Ti_3_C_2_T_x_ enjoys focused research on surface passivation, encapsulation, and composite integration strategies that improve its stability and lifespan. While V_2_CT_x_ shows promising sensitivity, it is susceptible to rapid oxidation and structural decline, limiting its use without protective coatings.

### 4.5. Gas Sensing Mechanisms

The gas sensing abilities of MXenes are largely determined by their surface chemistry, electrical properties, and interactions with gas molecules. Primarily, MXene-based gas sensors function using chemiresistive sensing, where gas molecules’ adsorption causes changes in the electrical conductivity of the MXene [116]. When gas molecules transfer a charge to the MXene, there is a change in carrier concentration within the material [117]. The extent and direction of the conductivity change are affected by the type of gas and its charge transfer properties, particularly whether the gases act as electron donors or acceptors [118]. The surface terminations of MXenes include –OH, –O, –F, and –Cl groups, which establish hydrogen bonds or other interactions when absorbing polar gases and volatile organic compounds (VOCs) [32]. The synthesis process controls the arrangement and density of these functional groups, thereby improving the detection capabilities of certain MXenes. Beyond electrical transduction, MXenes can also function in mass-sensitive sensors such as micro quartz tuning fork (MQTF) devices. In this context, gas molecule adsorption alters the mass of the MXene-coated sensor, resulting in a noticeable shift in resonance frequency. This approach enables gas sensing regardless of the inherent conductivity of MXenes and allows for enhanced chemical modifications to boost selectivity [116]. Combining MXenes with other nanomaterials, such as metal oxides and polymers, in heterostructures or composites can improve gas sensing capabilities. This improvement is realized by increasing the number of active sites, boosting selectivity, and encouraging synergistic effects [119]. In addition, MXenes’ large surface-to-volume ratio offers numerous active sites for gas adsorption, improving sensitivity. The gas sensing response may vary with layer thickness; generally, monolayer or few-layer MXenes display greater sensitivity because of their more accessible surface sites. By functionalizing them with specific chemical groups or doping, one can adjust the interaction strength between MXenes and target gases, thereby optimizing their selectivity and response time. Typically, MXenes are operated at room temperature to avoid surface oxidation, which could compromise their long-term stability and sensing performance [120]. MXenes are versatile sensing materials for the detection of gases, as they integrate excellent electronic properties with extensive surface functionalities and flexible detection techniques. By employing surface engineering and forming composites or developing heterostructures, the performance of MXenes can be significantly advanced, paving the way for next-generation sensors [121].

**Figure 10:**
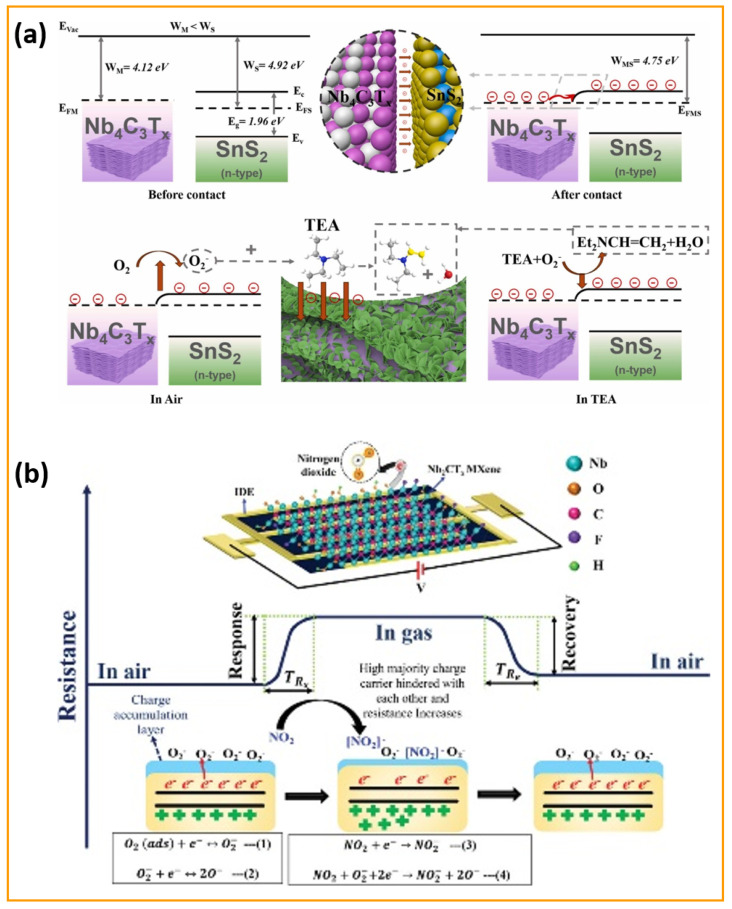
(**a**) Schematic illustration of the gas sensing mechanism of the SnS_2_/Nb_4_C_3_T_x_ composite toward triethylamine (TEA). The mechanism involves a charge transfer from Nb_4_C_3_T_x_ to SnS_2_ due to differences in work function, the formation of surface-bound O_2_^−^ species in air, and subsequent redox reactions with TEA that release electrons, reduce the depletion layer, and lower sensor resistance, resulting in a strong chemiresistive response at room temperature. Reproduced with permission from Ref. [100]. Copyright (2024) Elsevier. (**b**) Schematic illustration of the gas sensing mechanism of delaminated Nb_2_CT_x_ MXene functionalized with cetyltrimethylammonium bromide (CTAB) for NO_2_ detection. Upon exposure to air, oxygen molecules are adsorbed on the Nb_2_CT_x_ MXene surface and ionized into O_2_− species, forming an electron depletion layer by withdrawing electrons from the conduction band. When exposed to NO_2_ gas, an additional electron withdrawal occurs, leading to a further increase in resistance due to holes’ accumulation. The CTAB functionalization enhances interlayer spacing, surface area, and stability, enabling improved gas diffusion, selective adsorption, and reduced degradation under ambient conditions. The combination of surface functional groups (–O, –OH, –F), intercalated CTAB, and the metallic conductivity of Nb_2_CT_x_ collectively contributes to the enhanced sensitivity and signal-to-noise ratio of the sensor. Reproduced with permission from Ref. [122]. Copyright (2022) Wiley-VCH GmbH.

Figure 10a illustrates the proposed gas sensing mechanism of the SnS_2_/Nb_4_C_3_T_x_ composite sensor toward triethylamine (TEA), highlighting the synergistic interaction between the semiconducting SnS_2_ and the metallic Nb_4_C_3_T_x_ MXene [100]. Upon the formation of the heterostructure, electrons are transferred from Nb_4_C_3_T_x_ (with a lower work function of 4.12 eV) to SnS_2_ (work function of 4.92 eV) until Fermi level equilibrium is achieved, resulting in the formation of a depletion layer at the SnS_2_/Nb_4_C_3_T_x_ interface. When the SnS_2_/Nb_4_C_3_T_x_ sensor is exposed to ambient air, oxygen molecules are adsorbed and ionized to form O_2_^−^ species by capturing electrons from the SnS_2_ conduction band, thereby increasing the SnS_2_/Nb_4_C_3_T_x_ sensor’s resistance. The introduction of TEA, a strong reducing gas, initiates a redox reaction with the surface-bound O_2_^−^, leading to the release of electrons back into the conduction band and the narrowing of the depletion layer, which significantly reduces the SnS_2_/Nb_4_C_3_T_x_ sensor’s resistance. This process is supported by a sequence of reaction steps involving C–H bond dissociation in TEA, the formation of hydroxyl species, and the generation of stable intermediates such as EtNC = CH_2_ and water. The enhanced sensing response is attributed to the high surface area provided by in situ grown SnS_2_ nanosheets, the conductive nature of Nb_4_C_3_T_x_ facilitating efficient charge transport, and the strong electronic affinity of TEA for the composite surface. Figure 10b illustrates the proposed gas sensing mechanism underlying the enhanced chemiresistive performance of CTAB-delaminated Nb_2_CT_x_ (Nb_2_CT_x_-CTAB) MXene toward nitrogen dioxide (NO_2_) detection [122]. The mechanism is predominantly governed by surface charge interactions facilitated through functional groups such as –O, –OH, and –F, which remain on the surface of the delaminated Nb_2_CT_x_ MXene following hydrofluoric acid treatment and subsequent surfactant-assisted exfoliation. Upon exposure to atmospheric oxygen, the Nb_2_CT_x_-CTAB sensor surface adsorbs oxygen molecules, which are subsequently ionized into O_2_^−^ species through electron abstraction from the Nb_2_CT_x_ conduction band. This process forms an electron depletion layer and modulates the sensor’s baseline conductivity. The introduction of NO_2_, a strong electron-withdrawing oxidizing gas, further intensifies the depletion effect by capturing additional electrons from the Nb_2_CT_x_-CTAB sensing layer. This results in a noticeable increase in resistance, characteristic of p-type sensing behavior. The Nb_2_CT_x_-CTAB sensor’s responsiveness is directly attributed to the availability of active sites and functional groups that facilitate gas adsorption and electron transfer. The CTAB surfactant plays a dual role, such as (i) it promotes the effective delamination and interlayer swelling of Nb_2_CT_x_, increasing the surface area and enhancing gas diffusion; and (ii) it contributes to environmental stability by forming a hydrophobic barrier that inhibits oxidative degradation. The presence of CTA^+^ cations may also assist in tuning the surface potential and functional group orientation, further optimizing the interaction with target gas molecules. Moreover, the metallic conductivity inherent to Nb_2_CT_x_ ensures a low electrical noise, which is crucial for preserving a high signal-to-noise ratio during NO_2_ gas detection. This conductivity, combined with the structural and chemical modifications induced by CTAB, accounts for the superior sensitivity and response–recovery characteristics observed in the Nb_2_CT_x_-CTAB sensor compared to its pristine Nb_2_CT_x_. The mechanistic pathway depicted in Figure 10b thus corroborates the empirical findings presented in the study. It substantiates the potential of functionalized Nb_2_CT_x_ MXenes for next-generation gas sensing technologies, especially under ambient and humid conditions.

## 5. Conclusions and Future Prospects

Nb_2_CT_x_ MXene has emerged as an auspicious material in evolving advanced sensing platforms, including gas sensors, electrochemical devices, wearable biosensors, and environmental monitoring systems. Its exceptional properties, such as a high electrical conductivity, tunable surface chemistry, and excellent mechanical flexibility, facilitate the precise detection of trace chemical markers and hazardous substances while enabling reliable environmental monitoring. Despite these advantages, several critical challenges persist; protecting Nb_2_CT_x_ MXene from oxidation, developing scalable and reproducible synthesis methods, and effectively integrating the Nb_2_CT_x_ MXene material into flexible and practical devices remain essential research priorities. Furthermore, a deeper understanding of the interactions between Nb_2_CT_x_ MXene and the target analytes is required to optimize its sensitivity and selectivity. Simultaneously, strategies to lower production costs, enhance device durability, and conduct comprehensive lifetime assessments of Nb_2_CT_x_ MXene are crucial for enabling real-life applications. Looking forward, the future of Nb_2_CT_x_ MXene research lies in its hybridization with advanced materials such as conductive polymers, metal nanoparticles, and biomimetics. These hybrid materials hold the potential to unlock novel functionalities and significantly improve Nb_2_CT_x_ MXene device performance. Currently, Nb_2_CT_x_ MXene stands at the forefront of sensor technology development, with multidisciplinary research actively pursuing innovative solutions to create highly sensitive, eco-friendly health monitoring and environmental sensing systems for clinical and industrial applications.

As a sustainable material for environmental monitoring, Nb_2_CT_x_ MXene offers considerable advantages. Its high surface area, adaptable surface chemistry, and robust electrical properties enable the effective detection of various environmental pollutants. The Nb_2_CT_x_ MXene material’s rapid response and recovery times make it highly suitable for real-time environmental monitoring, allowing for proactive pollution management before contamination levels become critical. Future research should prioritize green chemistry approaches, integrating Nb_2_CT_x_ MXene with biodegradable materials to create sustainable, eco-friendly sensing platforms. Rigorous investigations in this direction could lead to developing advanced air quality monitoring systems, contributing meaningfully to global environmental protection efforts. In the biomedical domain, Nb_2_CT_x_ MXene exhibits tremendous potential due to its superior biocompatibility, outstanding electrical properties, and versatile surface functionalities. These attributes position Nb_2_CT_x_ MXene as an ideal candidate for the sensitive detection of biomolecules at early disease stages, thereby improving diagnostics and facilitating personalized health monitoring. Adding Nb_2_CT_x_ MXene into wearable and implantable medical devices could enable continuous, real-time health surveillance. However, to fully harness its biomedical potential, it is essential to address challenges such as elucidating biomolecular interaction mechanisms, ensuring long-term biocompatibility, and establishing standardized fabrication protocols. Collaborative efforts among clinical researchers, materials scientists, and biomedical engineers will be pivotal in overcoming these hurdles. Nb_2_CT_x_ MXene thus emerges as an innovative material poised to revolutionize environmental and biomedical sensing technologies. Through continued interdisciplinary research and technological advancement, Nb_2_CT_x_ MXene can significantly enhance sensors’ performance and ultimately contribute to improved human health and ecological sustainability.

Future studies will likely focus on mixing Nb_2_CT_x_ MXene with other advanced materials to enhance its sensing performance. Hybridization with conductive polymers, metal nanoparticles, metal–organic frameworks (MOFs), carbon nanotubes, and biomimetic substances can dramatically increase its sensitivity, selectivity, and stability. These multifunctional hybrids could enable the simultaneous detection of diverse analytes and operate in complex environments such as polluted atmospheres. The future of materials science is closely tied to sustainability objectives. Developing eco-friendly, low-energy, and scalable synthesis routes for Nb_2_CT_x_ MXene is imperative. Research should aim to minimize the use of hazardous etchants by exploring greener alternatives, including electrochemical or biologically mediated etching processes. Furthermore, incorporating Nb_2_CT_x_ MXene with biodegradable substrates and eco-friendly polymers could create fully sustainable, disposable, and environmentally benign sensor devices. While Nb_2_CT_x_ MXene demonstrates an excellent initial performance, its long-term stability, particularly in ambient and aqueous environments, requires significant improvement. Strategies such as surface functionalization, protective coatings, or encapsulation techniques may be employed to mitigate oxidation and degradation. In-depth studies of degradation mechanisms at the molecular level will be crucial to designing robust, long-lasting sensors suitable for real-world applications. The intrinsic mechanical flexibility of Nb_2_CT_x_ MXene makes it an ideal candidate for use in flexible electronics. Future advancements are expected to see Nb_2_CT_x_ MXene integrated into wearable health monitoring systems and implantable biosensors capable of continuous, real-time physiological monitoring.

In the future, sensor networks based on Nb_2_CT_x_ MXene could form the foundation of intelligent healthcare and environmental management ecosystems. For Nb_2_CT_x_ MXene-based sensors to achieve commercial success, standardized fabrication processes, rigorous testing protocols, and clear regulatory frameworks must be established. Collaboration between academia, industry, and regulatory bodies is essential to accelerate the translation of laboratory-scale innovations into market-ready products. Additionally, developing cost-effective manufacturing techniques will ensure broad accessibility and applications across multiple sectors. The future success of Nb_2_CT_x_ MXene lies at the intersection of numerous scientific disciplines. Close collaboration among chemists, materials scientists, biomedical engineers, clinicians, and environmental researchers will be vital to overcoming its current limitations. Interdisciplinary efforts will help unlock novel applications, from point-of-care diagnostic tools to sophisticated ecological monitoring networks. Compared to conventional gas sensing nanomaterials such as metal oxides, carbon, and conducting polymers, Nb_2_CT_x_ MXene presents a significant advancement by addressing critical bottlenecks that have historically limited sensors’ efficiency. Conventional nanomaterials often suffer from poor selectivity, slow response/recovery times, high operating temperatures, and limited flexibility. In contrast, Nb_2_CT_x_ MXene offers exceptional metallic conductivity for ultrafast electron transport, tunable surface terminations for improved analyte specificity, and intrinsic flexibility for integration into next-generation wearable platforms under ambient conditions. These unique features enable not only more sensitive and selective detection but also the design of low-power, real-time monitoring systems suitable for both clinical and environmental perspectives. Thus, Nb_2_CT_x_ MXene does not merely add to the existing pool of sensing materials but redefines the sensing paradigm by offering a multifaceted solution to long-standing material and application challenges. In summary, Nb_2_CT_x_ MXene emerges not just as a promising material but also as a disruptive platform capable of redefining the future of sensor technology through its ability to overcome the critical limitations of traditional materials and enable next-generation biomedical and environmental sensing solutions.
